# Open-access multidistance fNIRS dataset generated during ball-squeezing and motion-artifact induction tasks

**DOI:** 10.1117/1.NPh.13.S3.S32606

**Published:** 2026-10-22

**Authors:** Shibo Zhou, De’Ja Rogers, David A. Boas, Meryem A. Yücel, Yuanyuan Gao

**Affiliations:** a Wichita State University, Biomedical Engineering Department, Wichita, Kansas, United States; b Boston University, Neurophotonics Center, Boston, Massachusetts, United States

**Keywords:** multidistance functional near-infrared spectroscopy, diffuse optical tomography, open-access dataset, ball-squeezing, resting, motion artifact generation

## Abstract

**Significance:**

Although multidistance functional near-infrared spectroscopy (fNIRS) features higher spatial resolution and depth specificity compared with sparse fNIRS arrays, no open-access multidistance fNIRS dataset is available for motor and motion-artifact induction tasks.

**Aim:**

In this data descriptor, we introduce an open-access, multidistance fNIRS dataset to facilitate algorithm development and validation.

**Approach:**

The dataset was collected from 12 healthy young adults using a multidistance probe placed over the bilateral motor cortex. The experimental protocol included resting state task, ball-squeezing task, and motion-artifact induction task. Signal quality and motion artifact were quantitatively assessed using signal-to-noise ratio (SNR), motion artifact ratio (MAR), channel pruning statistics, and coefficient of variation (c.v.) across shorter (19 mm) and longer (32.9 mm) channels. The motion-artifact induction task was characterized using percent signal change, HbO/HbR concentration changes, and accelerometer data across six motion types.

**Results:**

Both resting and ball-squeezing tasks induced higher SNR and lower MAR than motion-artifact induction task. Shorter channels exhibited significantly higher SNR than longer channels during resting state. Unilateral ball-squeezing motion-induced oxy-hemoglobin increases in the contralateral motor cortex. Tilting up produced the largest and saying hello produced the smallest artifact across the six motion types. Head-tilting motions produced larger accelerometer changes than facial motions.

**Conclusions:**

We expect that this open-access dataset will accelerate the development and validation of algorithms specifically designed for multidistance fNIRS.

## 1 Introduction

Functional near-infrared spectroscopy (fNIRS) is a noninvasive, portable, and relatively low-cost optical neuroimaging technique.^1^ It measures changes in hemoglobin concentration to infer brain activity. Standard fNIRS typically uses "sparse" arrays, where source–detector separations (SDSs) are fixed at typically 30 mm. However, these sparse configurations are limited by low spatial resolution and a lack of depth specificity, making it difficult to distinguish cortical signals from superficial physiological noise in the scalp and skull. To address these issues, diffuse optical tomography (DOT) employs overlapping channels with multiple SDS.^2^^,^^3^ This multidistance fNIRS strategy enables reconstructing detailed three-dimensional (3D) maps of hemoglobin concentration changes to achieve higher spatial resolution and depth specificity compared with sparse fNIRS.^4^ High-density DOT (HD-DOT) uses “dense” arrays to increase the imaging performance of DOT, particularly in image resolution and localization.^5^

Previous studies have validated HD-DOT across diverse domains. Studies utilizing paradigms ranging from retinotopic mapping and tactile stimulation^6^^,^^7^ to complex cognitive tasks^8^^–^^10^ have consistently demonstrated HD-DOT’s capacity to map cortical activity with high localization accuracy. In addition, taking advantage of predictable spatial activation patterns generated by motor tasks—unilateral movements consistently producing contralateral motor cortex activation,^3^^,^^11^ motor tasks have also been used to validate HD-DOT image reconstruction. For example, simple finger-to-thumb touching,^11^ vibrotactile stimulation,^12^ finger–thumb opposition,^13^ and arm imitation^14^ tasks have demonstrated the capacity of HD-DOT to localize cortical activation. However, the datasets generated from these studies are not open-access.

Existing open-access motor-task fNIRS datasets include motor imagery,^15^ unilateral finger- and foot-tapping,^16^ and leg movement.^17^ These datasets are valuable for motor-task classification and channel-based analysis, but they are primarily based on sparse fNIRS arrays, which lack the channel overlap preferred for 3D image reconstruction and depth specificity. To address these limitations, an open-access multidistance fNIRS dataset with motor task is needed.

In parallel, motion-artifact detection and removal methods have been suggested and tested for the standard fNIRS, including spline interpolation,^18^ wavelet-based filtering,^19^ targeted principal component analysis,^20^ and temporal derivative distribution repair.^21^ Tasks designed to introduce controlled motion artifacts, such as head^22^^,^^23^ and jaw^24^ movements, have also been used to characterize motion artifacts in fNIRS. However, there is no such method or task specified for multidistance fNIRS arrays. Even though traditional HD-DOT systems are large in size and not portable,^11^ our previous work^25^ has demonstrated a light-weight multidistance fNIRS using two portable fNIRS devices. However, this portability will induce more motion artifacts and require specific motion artifact removal technologies. To test such motion artifact removal methods, a multidistance fNIRS dataset with controlled motion artifacts is needed.

Identifying the two gaps, we propose to introduce an open-access fNIRS dataset with a light-weight, multidistance montage design. This dataset includes three tasks: (1) resting state, (2) ball-squeezing, and (3) motion-artifact induction tasks. The data are formatted according to the BIDS standard^26^^,^^27^ to ensure ease of use and reproducibility. Our previous work^25^ used the ball-squeezing data in this dataset to validate DOT image reconstruction. The present study extends that work by publicly releasing the complete dataset and by characterizing signal quality, motion artifacts, and accelerometer data, as well as validating task-evoked brain activation. We have uploaded this dataset to the OpenNeuro platform^28^ to be open-access. This dataset could be potentially used by other researchers, for example, to test image reconstruction (e.g., regularization), signal processing (e.g., motion-correction), and brain–computer interface methods (left versus right hand ball-squeezing classification).

## 2 Method

### 2.1 Participants

This fNIRS dataset was collected from 12 healthy young adults (6 females/6 males, aged 23.2±5.2  years ranging from 18 to 37 years). Participant demographics are summarized in Table 1. The study protocol was approved by the Institutional Review Board of Boston University, and all participants provided written informed consent prior to data collection.

**Table 1:** Participant demographics (N=12).

Participant ID	Age	Gender	Race	Handedness	Ethnicity
170	21	Female	White	Right	Not Hispanic or Latino
171	Not Reported	Female	Asian	Left	Not Hispanic or Latino
173	37	Male	White	Right	Not Hispanic or Latino
174	23	Female	White	Right	Not Hispanic or Latino
176	25	Male	White	Right	Not Hispanic or Latino
177	25	Female	White	Right	Not Hispanic or Latino
179	20	Male	White	Right	Not Hispanic or Latino
181	23	Female	White	Right	Not Hispanic or Latino
182	18	Male	Asian	Right	Not Hispanic or Latino
183	24	Female	White	Right	Not Hispanic or Latino
184	19	Male	White	Right	Not Hispanic or Latino
185	20	Male	White	Left	Not Hispanic or Latino
Average	23.2±5.2	F: 6, M: 6	W: 10, A: 2	R: 10, L: 2	Not Hispanic or Latino: 12

### 2.2 fNIRS Measurements

We designed an overlapping multidistance fNIRS probe with 7 sources and 16 detectors, forming 50 measurement channels for each wavelength in each patch. We placed two patches over the left and right motor cortex regions, resulting in a total of 100 channels for each wavelength. To differentiate oxy- and deoxy-hemoglobin, we employed two wavelengths, 760 and 850 nm, resulting in a total of 200 measurement channels. We also placed two accelerometers at Cz and Pz locations to detect head movement. The shorter SDSs were 19 mm apart, and the longer ones were 32.9 mm apart. The configuration is illustrated in 2D probes [Fig. 1(a)], and 3D probes onto the cortical surface [Fig. 1(b)]. The shorter channels (19 mm), which include both superficial and cortical sensitivity, are not conventional short-separation channels (∼10  mm).^29^ To record fNIRS signal using this multidistance probe configuration, we employed two cascaded NIRSport2 16×16 devices (NIRx Medical Technologies LLC, Berlin, Germany). The probe was designed in AtlasViewer,^30^ and the cap was 3D printed using NinjaCap.^31^ To ensure consistent optode placement across participants, we measured the Cz position of the head using the standard 10 to 20 international EEG locations and aligned it with the Cz marker of the cap. To verify measurement sensitivity over the motor cortex, we utilized AtlasViewer to perform Monte Carlo simulations and get the spatial sensitivity profile [Fig. 1(c)].

**Fig. 1:**
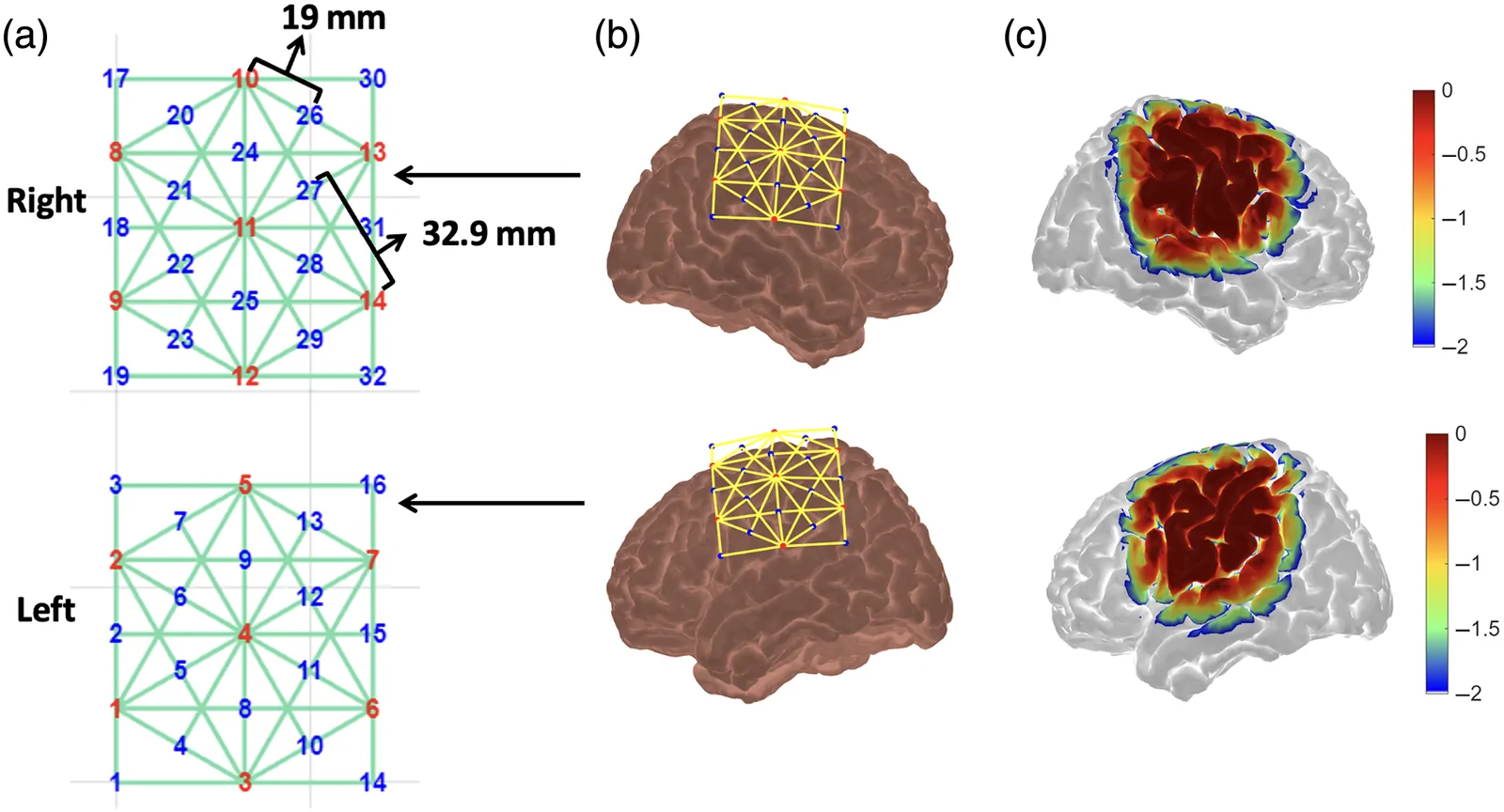
Probe configuration. (a) 2D layout of probe including sources, detectors, and measurement channels. Red numbers represent sources. Blue ones represent detectors. Green lines indicate measurement channels. (b) 3D co-registration of the probe onto the brain atlas. Yellow lines represent the spatial arrangement of the channels relative to the underlying brain areas. (c) The sensitivity profile: projected onto both left and right brain, with red colors representing high sensitivity to hemodynamic changes and blue colors representing low sensitivity. The color bar has sensitivity values on a log scale. The images were generated using AtlasViewer.^30^.

### 2.3 Experimental Paradigm

The participants made one-time visit to the lab lasting about 1 h. At the beginning of the visit, they were consented and instructed on three tasks, including resting state, ball-squeezing, and motion-artifact induction. They went through all three tasks with fNIRS recorded during each task. No head restraint (e.g., chin rest) was used. The participants first completed one 5-min run of resting state task, where they remained mentally idle, avoiding any head movement [Fig. 2(a)]. For the ball-squeezing task, the participants held a soft rubber ball (2.5 in. in diameter) in each hand and sat in front of a 13.3-in. computer screen. They squeezed the ball when a visual cue (the text “left” or “right”) appeared on the screen accompanied by an audio sound saying “left” or “right.” The stimulus lasted 1 s followed by a randomized interstimulus interval (ISI) of 5 to 15 s. Three runs of this ball-squeezing task were conducted, with 15 left-hand trials and 15 right-hand trials in each run in a fully randomized order [Fig. 2(b)]. For the motion-artifact induction task, the participants performed six movements to intentionally introduce controlled motion artifacts into the fNIRS signals, including tilting the head right, left, up, and down, lifting eyebrows, and saying hello.^32^ A visual cue for each trial appeared on the screen for 1 s with a corresponding audio cue, followed by a randomized ISI of 5 to 10 s [Fig. 2(c)]. They performed one run with 30 trials in total (five repetitions for each movement), presented in a fully randomized order.

**Fig. 2:**
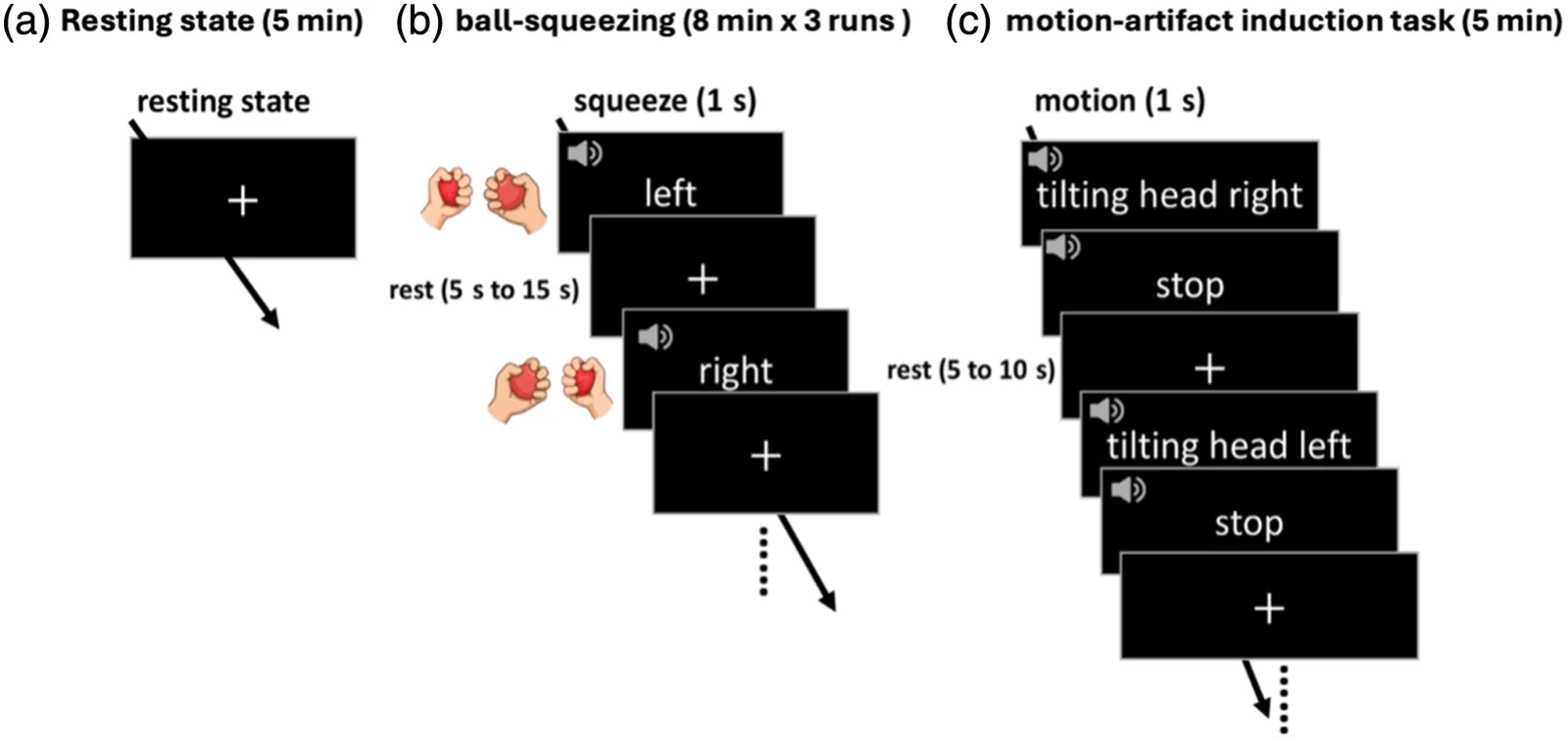
Experimental design. (a) resting state task: Participants were instructed to minimize head movement while fixing their gaze on a central cross for 5 min. (b) ball-squeezing task: The task consisted of 3 runs (30 trials per run). In each trial, a visual cue (“left” or “right”) was displayed for 1 s and accompanied by an audio command, followed by a randomized ISI of 5 to 15 s. (c) Motion-artifact induction task: Participants performed six distinct instructed movements (tilting head right/left/up/down, lifting eyebrows, and saying “hello”) to introduce controlled motion artifacts. The task included one run with 30 trials (five repetitions per movement). Each visual instruction appeared for 1 s with an audio cue, followed by a randomized ISI of 5 to 10 s.

## 3 Data Organization

We used BfNIRS Cloud platform^26^ to organize the raw fNIRS dataset into BIDS-compliance format. The directory hierarchy is structured by subject (e.g., sub-170/). At the root level of each session’s directory (e.g., sub-170/ses-01), a .tsv file provides the acquisition timing for each recording. fNIRS imaging data are stored under the nirs/subdirectory, which contains both the spatial geometry of the probe and the time-series data for each experimental run. Raw light intensity measurements are stored in the Shared Near Infrared File Format (*.snirf), which is a standard file format for fNIRS data.^33^ From each SNIRF file, the BfNIRS platform generates a _nirs.json file containing metadata about the NIRS system, including the task name, sampling frequency, number of channels, and the number of source and detector optodes; a _coordsystem.json file containing the coordinate reference frame and units; a_optodes.tsv file containing 3D positions of all sources and detectors, with a corresponding_optodes.json file defining the meaning of each column; a _channels.tsv file containing the measurement channel information, including source–detector pairings, nominal wavelengths, and units; a _events.tsv file logging the timing of events, including onset, duration, event type, and marker value. The event file is fully described by the accompanying_events.json file, which defines the meaning of each column. The dataset contains 60 SNIRF files (12 participants × 5 runs: one resting state run, three ball-squeezing runs, and one motion-artifact induction run per participant). Including all metadata files, the dataset contains 459 files in total.

To confirm BIDS compliance, the dataset was validated using the BIDS Validator (v3.0.0-alpha.3).^34^ The validator returned no errors across all 459 files. Warnings are detailed in the Supplementary Material.

## 4 Data Analysis

To validate the dataset and provide a reference for other users, we performed qualitative and quantitative assessment of signal quality and motion artifacts. The validation pipeline was implemented using custom MATLAB (R2025a, The MathWorks, Inc., Natick, Massachusetts) code with functions from Homer3 toolbox.^35^

### 4.1 Qualitative Assessment of the Signal

Qualitative assessment was performed by visually inspecting time-series raw signal.

First, we plotted the raw signal to verify the presence of cardiac pulsations. Robust and consistent cardiac oscillations indicate acceptable signal-to-noise ratio (SNR).^36^

Second, to verify whether the movements in motion-artifact induction task effectively induced motion artifacts, we plotted time-series raw light intensity data with the timing of the stimulus marked, to display the artifacts caused by motion-artifact induction task.

Finally, we plotted the accelerometer data to confirm that motion artifacts were induced by physical head movements.

### 4.2 Noise Level Analysis

Signal quality was computed directly on the raw light intensity data without applying any noise reduction or filtering algorithms. We used channel-wise signal-to-noise ratio (SNR) values, calculated as:

$${\mathrm{SNR}}_{\mathrm{dB}}=20\times \log_{10}(\frac{\mu }{\sigma }),$$ (1)

where μ is the mean signal intensity and σ is the standard deviation of the raw light intensity. For each task, the SNR values were averaged across channels grouped by shorter and longer channel distance, to account for different SNRs induced by the channel distance (19 and 32.9 mm).

We further quantified the motion artifacts by calculating the motion artifact ratio (MAR). MAR was performed on optical density data, which was converted using the hmrR_Intensity2OD function in the Homer3 toolbox from the raw light intensity data. For each channel, the MAR (%) was defined as the percentage of time points classified as artifacts relative to total recording duration:

$$\mathrm{MAR}(\%)=\frac{N_{\text{artifact}}}{N_{\text{total}}}\times 100,$$ (2)

where $N_{\text{artifact}}$ is the number of time points contaminated by motion artifacts calculated using the Homer3 hmrR_MotionArtifactByChannel function (tMotion=0.5  s, tMask=0.5  s, STDEVthresh=20, AMPthresh=5); $N_{\text{total}}$ is the total number of time points. Similar to SNR analysis, the MAR values were averaged across channels grouped by shorter and longer channel distance (19 and 32.9 mm).

We hypothesized that (1) the motion-artifact induction task would exhibit the lowest, ball-squeezing the second lowest, and resting state task the highest SNR. Conversely, we expected the motion-artifact induction task would exhibit the highest MAR, compared with the other tasks. (2) The longer channel distance exhibits lower SNR than shorter channel distance during resting state.

For each participant, we obtained one SNR value and one MAR value in each channel for the resting state and motion-artifact induction tasks. To statistically compare across the three tasks, we averaged the SNR and MAR across the three runs in ball-squeezing task.

Then, to statistically evaluate the differences across the three tasks, we performed a one-way repeated-measures analysis of variance (RM-ANOVA) separately for the shorter (19 mm) and longer (32.9 mm) channels. The within-subject factor was conditions (resting, ball-squeezing, motion-artifact induction). Significant main effects were followed by Bonferroni-corrected pairwise comparisons to identify specific differences between conditions (resting versus ball-squeezing, resting versus motion-artifact induction, and ball-squeezing versus motion-artifact induction). Finally, to test the hypothesis regarding channel distance, we compared the SNR values between shorter and longer channels during resting state using paired-samples t-tests. Statistical significance was set at p<0.05. Effect sizes were reported as partial eta-squared ($\eta p^{2}$) for RM-ANOVA main effects and Cohen’s d for Bonferroni-corrected pairwise comparisons and paired-samples t-tests.

We further assessed channel quality by applying the Homer3 hmrR_PruneChannels function ($\text{dRange}=[0,1\times 10^{7}]$, SNRthresh=10, SDrange=[0.0,45.0]). This function excluded channels with μ/σ≤10, which corresponds to SNR≤20  dB under our definition [Eq. (1)], or equivalently, coefficient of variation (c.v.=σ/μ)≥0.1. We computed the c.v. for each channel before and after pruning, averaged across channels grouped by shorter and longer channel distance.

### 4.3 Functional Integrity of Ball-Squeezing Task

To validate the functional integrity of the dataset, we hypothesized that hemodynamic response would exhibit expected hemispheric lateralization patterns in the ball-squeezing task: Specifically, right-hand squeezing would elicit activation in the left motor cortex, whereas left-hand squeezing would activate the right motor cortex. We followed the same analysis methods in our previous work.^25^ The processing pipeline is described in the Supplementary Material S1.

### 4.4 Quantitative Assessment of Motion-Artifact Induction Task

To characterize the motion-artifact induction task for each of the six motion types, we quantified both optical signal and accelerometer changes. We adapted two optical signal metrics from Yücel et al.^32^: percent signal change (PSC) and oxy-hemoglobin (HbO) and deoxy-hemoglobin (HbR) concentration changes.

PSC was computed for each wavelength (760 and 850 nm):

$$\text{Signal Change}(\%)=|1-\frac{{\text{Signal}}_{\text{motion artifact}[0\;\text{to}\;2\;\sec]}}{{\text{Signal}}_{\text{baseline}[-2\;\text{to}\;0\;\sec]}}|\times 100,$$ (3)

where ${\mathrm{Signal}}_{\text{motion artifact}}$ and ${\text{Signal}}_{\text{baseline}}$ are the mean raw light intensity averaged from 0 to 2 s after and −2 to 0 s before motion onset, respectively. The PSC was averaged across channels grouped by shorter and longer channel distance.

To obtain HbO and HbR concentration changes, raw light intensity was first converted into changes in optical density using the hmrR_Intensity2OD function in Homer3. The changes in HbO and HbR concentrations were then obtained using the hmrR_OD2Conc function with a partial pathlength factor of 6.^37^^–^^39^ For each motion type, we computed the absolute mean HbO and HbR concentration changes from 0 to 2 s after motion onset, averaged across channels grouped by shorter and longer channel distance. We also computed the standard deviation of HbO and HbR concentration changes across the motion-artifact induction task duration for shorter and longer channels.

To quantify head movement for each motion type, we calculated the acceleration change at the Cz position for each axis (X, Y, Z):

$$\mathrm{ACC}\;\text{change}=|{\mathrm{ACC}}_{\text{motion artifact}[0\;\text{to}\;2\;\sec]}-{\mathrm{ACC}}_{\text{baseline}[-2\;\text{to}\;0\;\sec]}|,$$ (4)

where ${\mathrm{ACC}}_{\text{motion artifact}}$ and ${\mathrm{ACC}}_{\text{baseline}}$ are the mean acceleration averaged from 0 to 2 s after and −2 to 0 s before motion onset, respectively.

Finally, to examine the relationship between optical signal and acceleration changes, we calculated Spearman’s rank correlation coefficients between PSC and Cz acceleration changes for 12 combinations (2 wavelengths × 2 channel distances × 3 acceleration axes). Before calculating the correlations, PSC and acceleration change values were log-transformed and z-score normalized. Because each participant contributed six data points, one per motion type, the data points within each correlation were not independent. We therefore report the correlation coefficients descriptively.

## 5 Technical Validation

### 5.1 Qualitative Visual Check

First, the raw light intensity signals displayed consistent cardiac pulsations across the resting state, ball-squeezing, and motion-artifact induction tasks (Fig. 3), which indicated acceptable signal-to-noise ratio. Second, in the motion-artifact induction task, the light intensity displayed signal fluctuations immediately following task stimulus (Fig. 4). These fluctuations confirmed that the motion-artifact induction task effectively introduced the intended motion artifacts. Finally, the accelerometer time series showed spikes in the X, Y, and Z axes that were temporally aligned with the motion-artifact induction task stimulus (Fig. 5). Lifting eyebrows produced intensity changes (Fig. 4) but minimal accelerometer changes (Fig. 5). Intensity changes observed before some stimulus onsets reflect task-unrelated spontaneous movements rather than instructed motions.

**Fig. 3:**
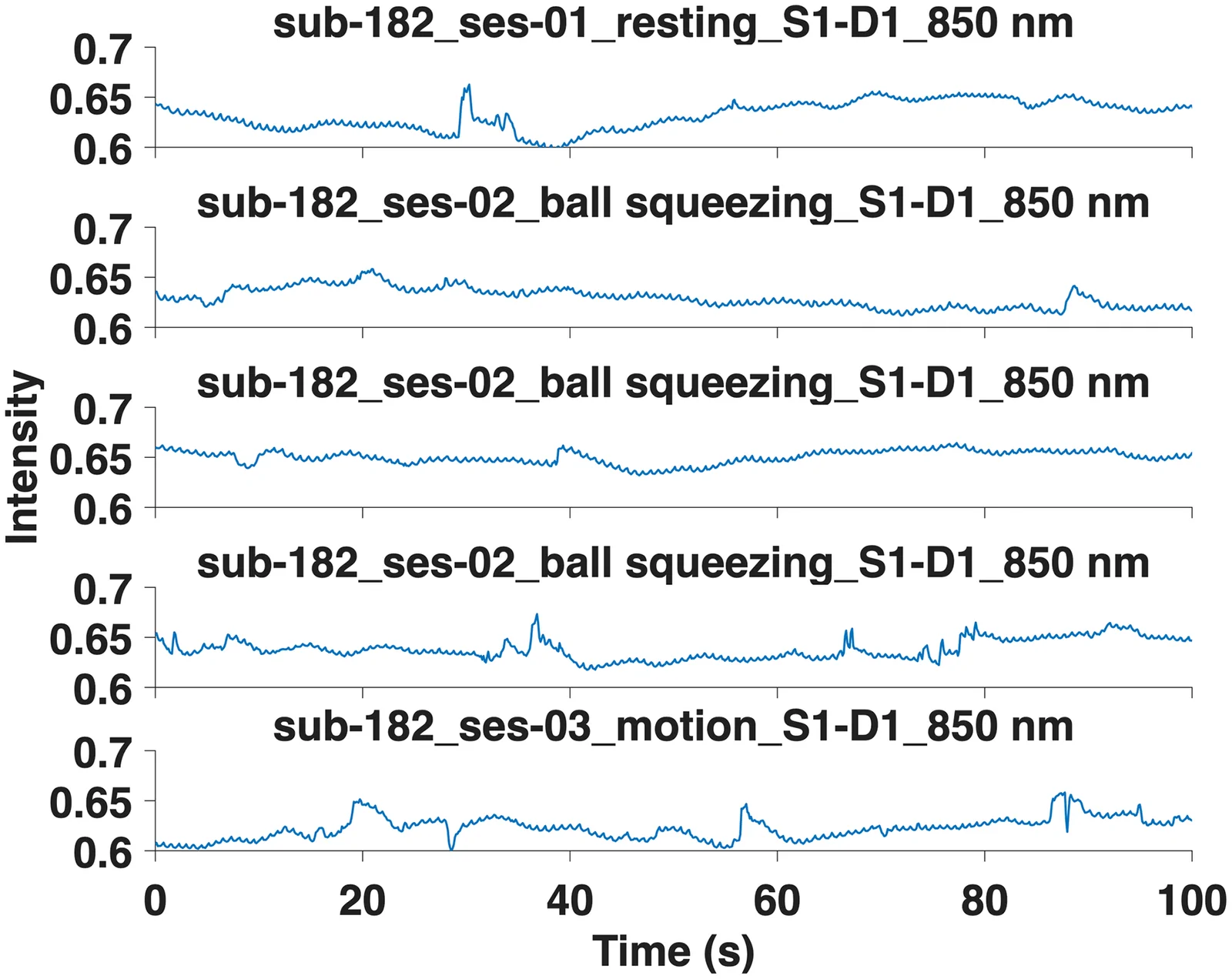
Raw fNIRS signals. Time-series data of raw light intensity from a representative subject (sub-182, source–detector pair S1-D1, 850 nm) across the three tasks in the first 100 s: resting state, three runs of ball-squeezing, motion-artifact induction task. Consistent cardiac pulsations are visible throughout the three tasks, indicating acceptable signal-to-noise ratio (SNR).

**Fig. 4:**
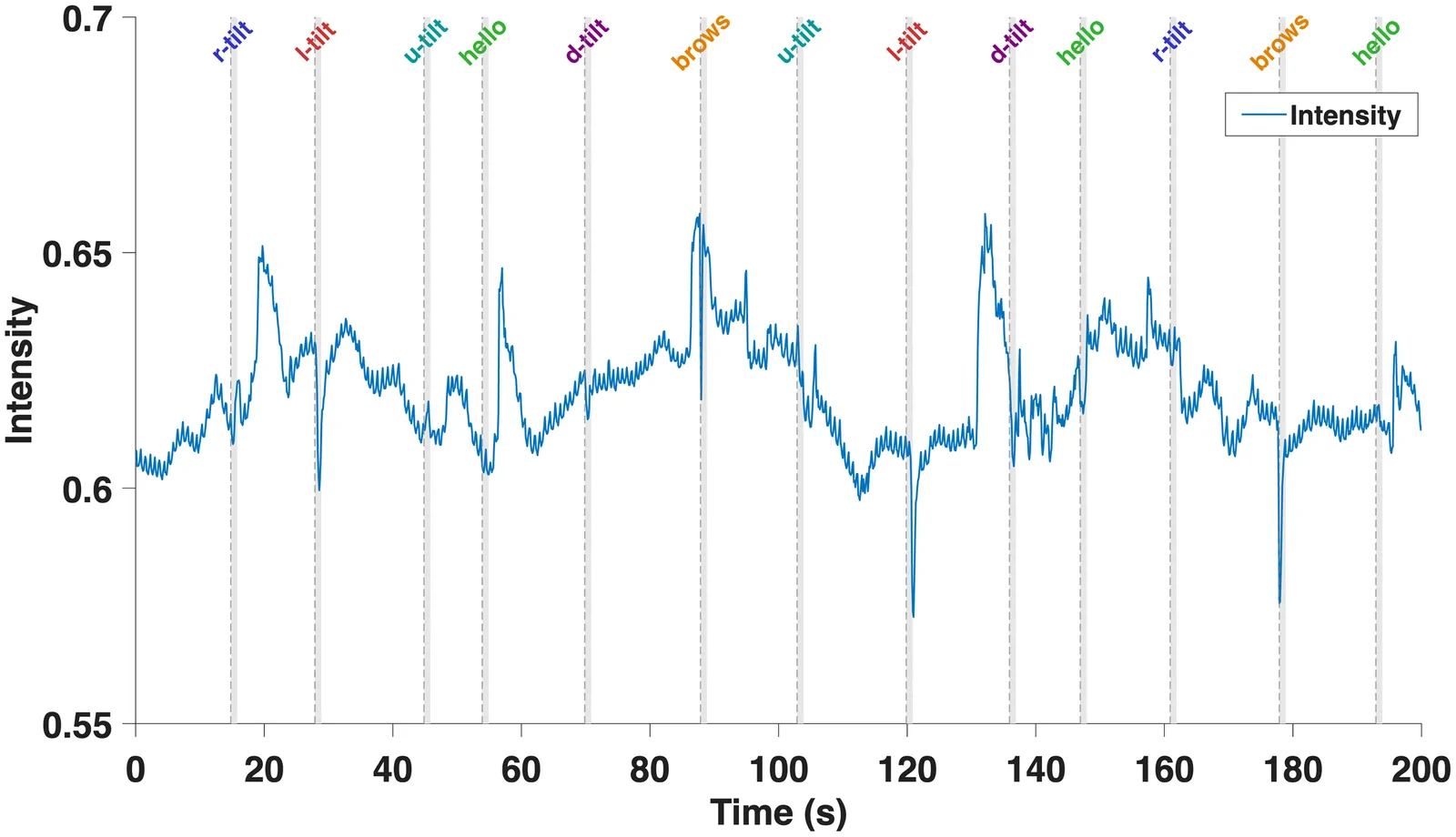
Raw light intensity during motion-artifact induction tasks. Time-series data from a representative subject showing signal responses to six instructed movements: tilting head up (u-tilt), left (l-tilt), down (d-tilt), right (r-tilt), saying "hello" (hello), and lifting eyebrows (brows). The plot displays 200 s recording window. Vertical dashed lines and gray shaded areas indicate the timing of the motion-artifact induction task stimulus and duration. Spikes of the time-series data are aligned with the task stimulus, confirming that the motion-artifact induction task effectively introduced the intended artifacts.

**Fig. 5:**
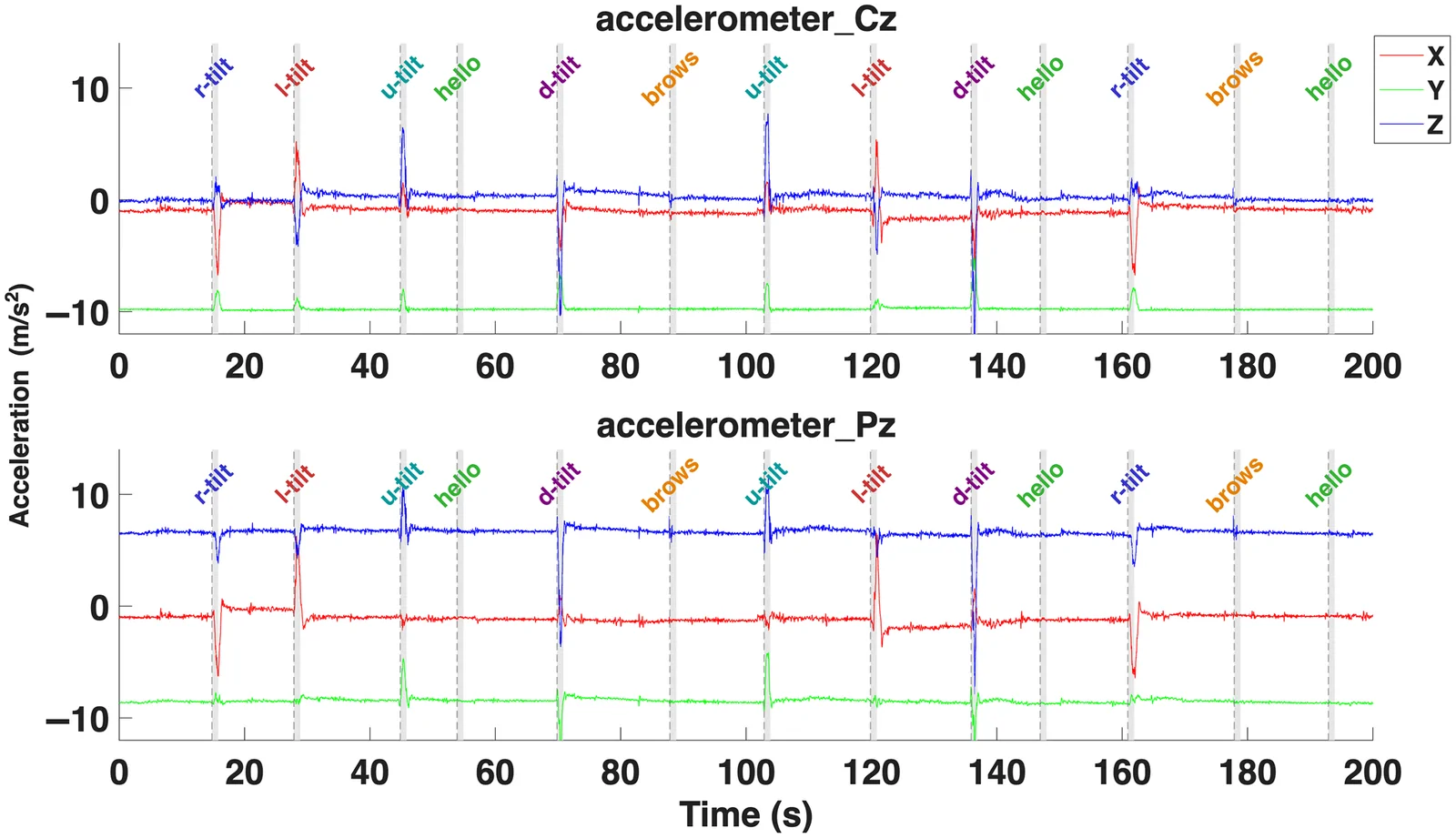
Accelerometer data during motion-artifact induction task. Acceleration recorded in the X (red), Y (green), and Z (blue) axes at the Cz (above) and Pz (below) locations. Vertical dashed lines and gray shaded areas indicate the timing of the motion-artifact induction task stimulus and duration. Spikes in the acceleration are aligned with the task stimulus, verifying that the motion-artifact induction task effectively induced head movements.

### 5.2 Noise Level Validation

To assess signal quality, we analyzed the signal-to-noise ratio (SNR) and motion artifact ratio (MAR) across the three tasks. Prior to statistical analysis, the normality of the data distribution was assessed using the Lilliefors test.^40^ Results indicated that although data in the ball-squeezing, and motion-artifact induction tasks were normally distributed (p>0.05), the resting state data exhibited deviations from normality (p<0.05). Given the robustness of ANOVA to violations of normality,^41^ parametric tests proceeded. Mauchly’s test indicated that the assumption of sphericity was violated for all four ANOVA models (SNR and MAR for both shorter and longer channels) (p<0.05); therefore, degrees of freedom were corrected using Greenhouse-Geisser estimates.^42^ The results are summarized in Fig. 6.

**Fig. 6:**
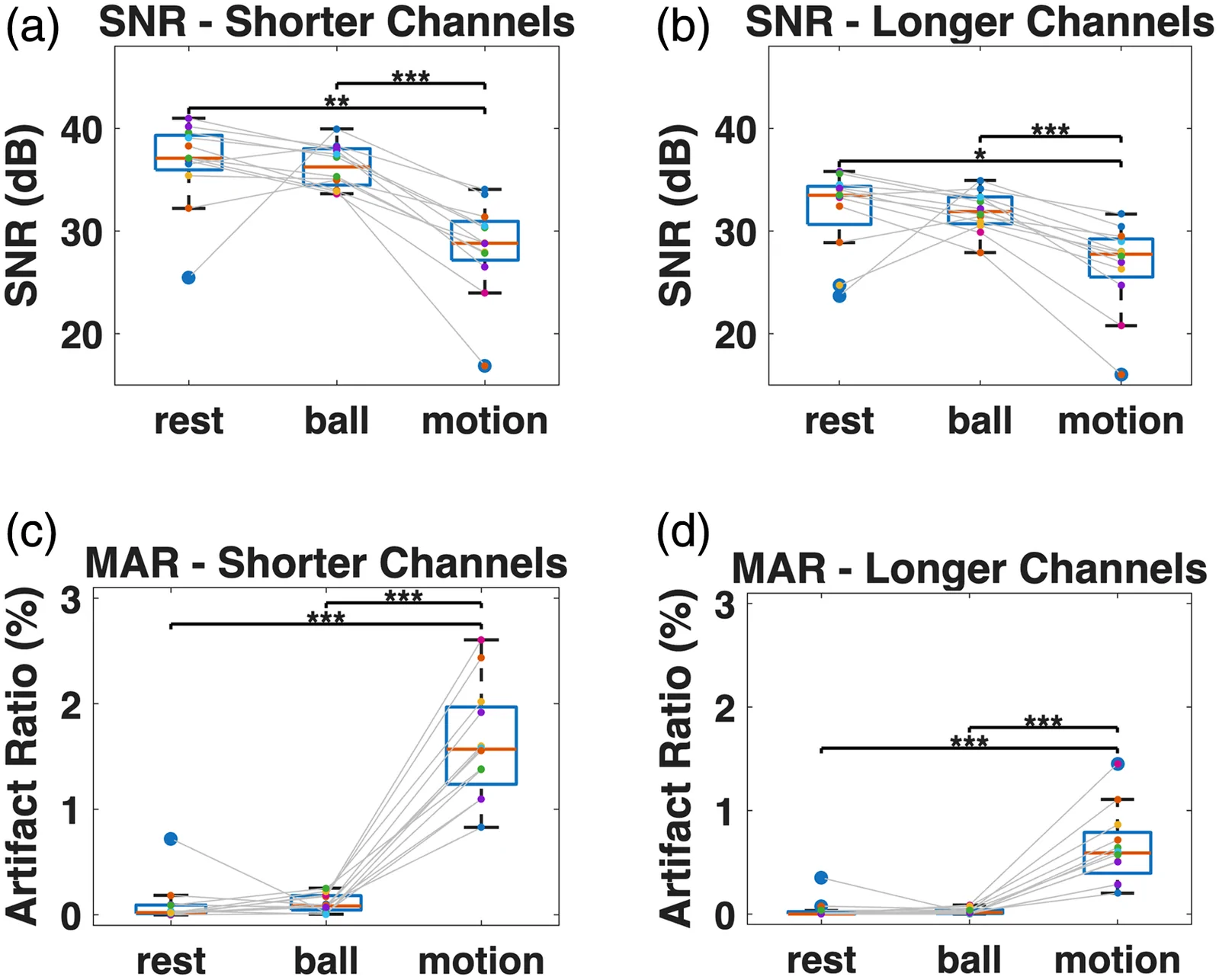
Comparisons of signal-to-noise ratio (SNR) and motion artifact ratio (MAR) across three tasks. Boxplots display the SNR (a) and MAR (b) for shorter (c) and longer (d) channels. SNR and MAR are compared across three experimental conditions: resting state, ball-squeezing, and motion-artifact induction task. The motion-artifact induction task exhibits significantly lower SNR and greater MAR compared with the resting state and ball-squeezing tasks, whereas no significant differences were observed between the resting state and ball-squeezing tasks. Individual participant data points are displayed with colored dots, and gray lines connect the same participant across tasks. Central red lines indicate median value, the upper and lower edges of the boxes represent the interquartile range (25th and 75th percentiles), the whiskers indicate the data range excluding outliers (defined as values extending beyond 1.5× the interquartile range), and the outliers are marked by large blue dots. Statistical significance between conditions is indicated by brackets and asterisks (*p<0.05, **p<0.01, ***p<0.001; Bonferroni-corrected).

In both shorter and longer channels, different tasks had a significant main effect in SNR (shorter channels: F(1.24,13.67)=16.62, p<0.001, $\eta p^{2}=0.602$; longer channels: F(1.22,13.4)=9.71, p<0.01, $\eta p^{2}=0.469$). Pairwise comparisons indicated that SNR in the motion-artifact induction task was significantly lower than in the resting state (shorter channels: p=0.008, d=1.107; longer channels: p=0.042, d=0.841) and ball-squeezing tasks (shorter channels: p<0.001, d=2.169; longer channels: p<0.001, d=1.790). There were no significant differences in SNR between the resting state and ball-squeezing tasks for either shorter or longer channel distance.

For MAR, different tasks had a significant main effect on both shorter and longer channels (shorter channels: F(1.18,12.97)=77.82, p<0.001, $\eta p^{2}=0.876$; longer channels: F(1.08,11.92)=31.26, p<0.001, $\eta p^{2}=0.740$). Pairwise comparisons indicated that MAR in the motion-artifact induction task was significantly higher than in the resting state (shorter channels: p<0.001, d=2.407; longer channels: p<0.001, d=1.508) and ball-squeezing tasks (shorter channels: p<0.001, d=3.010; longer channels: p<0.001, d=1.827). Consistent with the SNR results, no significant differences were found between the resting state and ball-squeezing task for either shorter or longer channel.

The normality of the difference in SNR between the shorter and longer channels during resting state was assessed using the Lilliefors test. The result indicated that the difference followed a normal distribution (p>0.05), satisfying the assumption of a paired samples t-test. Paired-samples t-tests between longer (32.9 mm) and shorter (19 mm) channels under resting state revealed significant differences in SNR (Fig. 7). To be specific, shorter channels exhibited significantly higher SNR compared with longer channels (t(11)=6.9, p<0.001, d=1.991) during resting state.

**Fig. 7:**
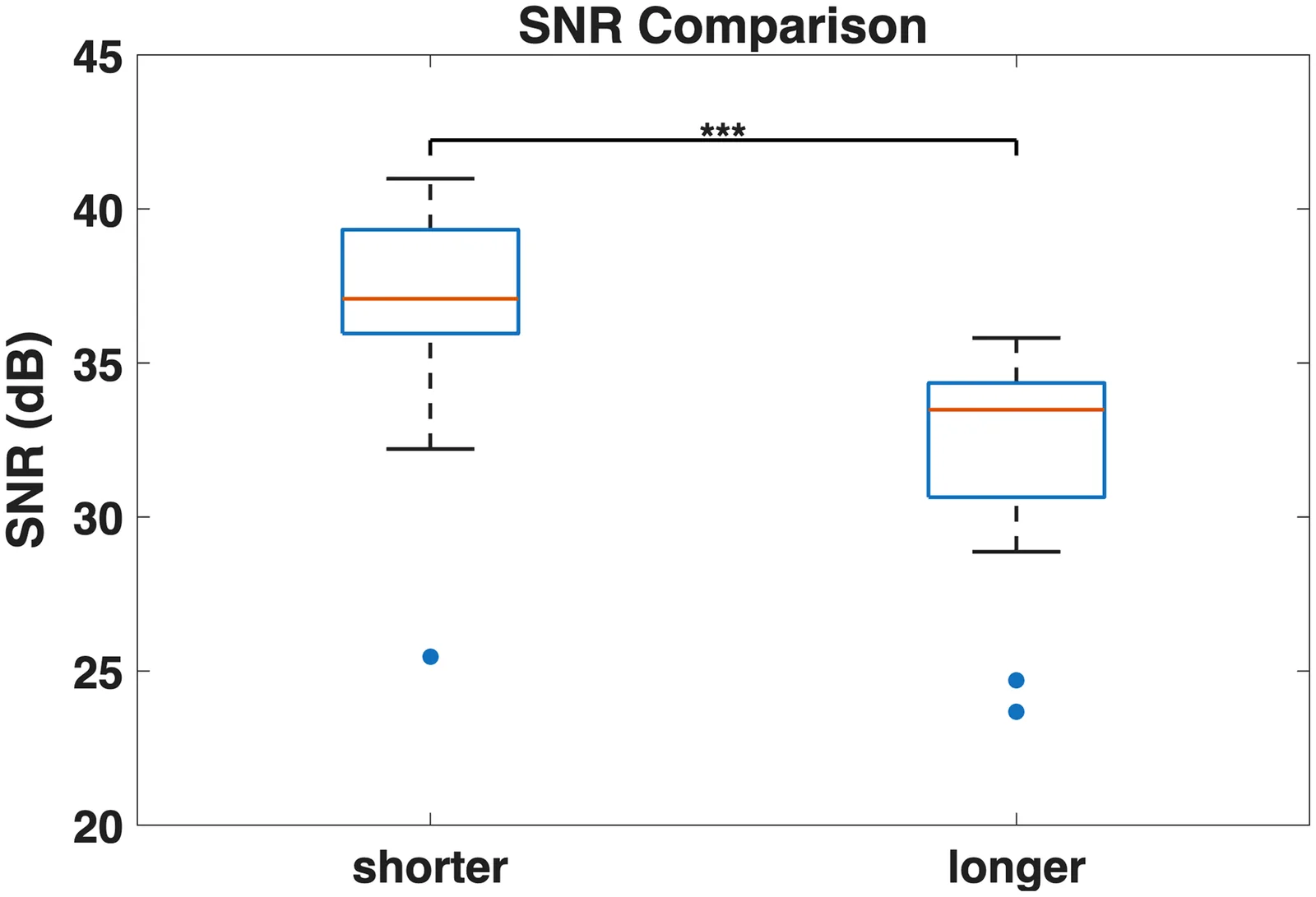
Comparison of SNR in resting state between channel distances. Paired t-test demonstrated that shorter channels exhibited significantly higher SNR compared with longer channels (p<0.001) during resting state. Central red lines indicate median value, the upper and lower edges of the boxes represent the interquartile range (25th and 75th percentiles), the whiskers indicate the data range excluding outliers (defined as values extending beyond 1.5× the interquartile range), and the outliers are marked by blue dots. Statistical significance between conditions is indicated by brackets and asterisks (*p<0.05, **p<0.01, ***p<0.001).

Finally, channel pruning statistics and c.v. values across tasks and channel distances are summarized in Fig. 8. Across all participants, the percentage of retained channels for shorter and longer channels was 99.11% and 95.83% during resting state, 99.70% and 97.92% during ball-squeezing, and 89.14% and 89.02% during the motion-artifact induction task. After pruning, c.v. values decreased across all tasks and channel distances. Additional channel-quality metrics are reported in the Figs. S3–S8 in the Supplementary Material.

**Fig. 8:**
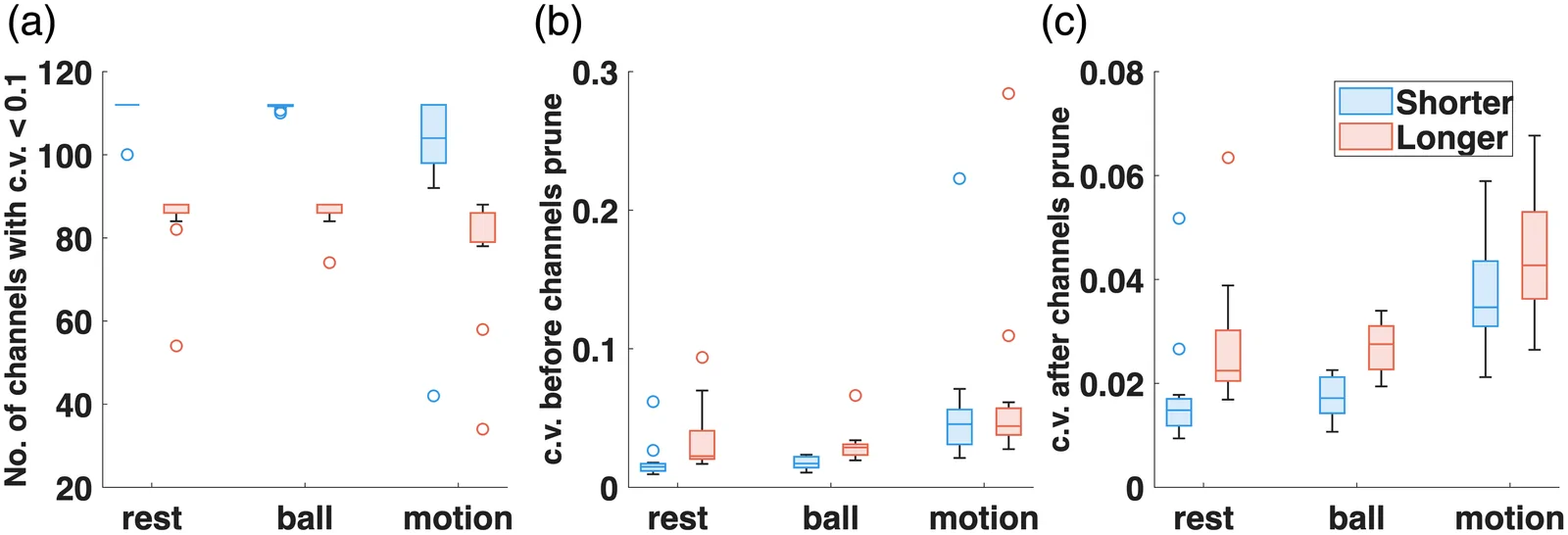
Channel pruning statistics and coefficient of variation (c.v.) across three tasks. (a) Number of channels with c.v.<0.1 for shorter (blue) and longer (red) channels across tasks. (b) c.v. values before channel pruning. (c) c.v. values after channel pruning. Central lines indicate median value, the upper and lower edges of the boxes represent the interquartile range (25th and 75th percentiles), the whiskers indicate the data range excluding outliers (defined as values extending beyond 1.5× the interquartile range), and the outliers are marked by dots.

### 5.3 Motor Lateralization Validation

Figure 9 presents the functional validation results for lateralization in the ball-squeezing task, reproduced from Gao et al. (2023) in their paper titled “Short-separation regression incorporated diffuse optical tomography image reconstruction modeling for high-density functional near-infrared spectroscopy,”^25^ using the same 12 participants and the same dataset. The results show the distinct contralateral pattern: squeezing the left hand elicited an increase in group average HbO concentration in the right motor cortex (contralateral hemisphere). Conversely, squeezing the right hand induced a corresponding group average HbO increase in the left motor cortex. The lateralization indicates that the ball-squeezing task induces the expected HbO responses associated with motor execution.

**Fig. 9:**
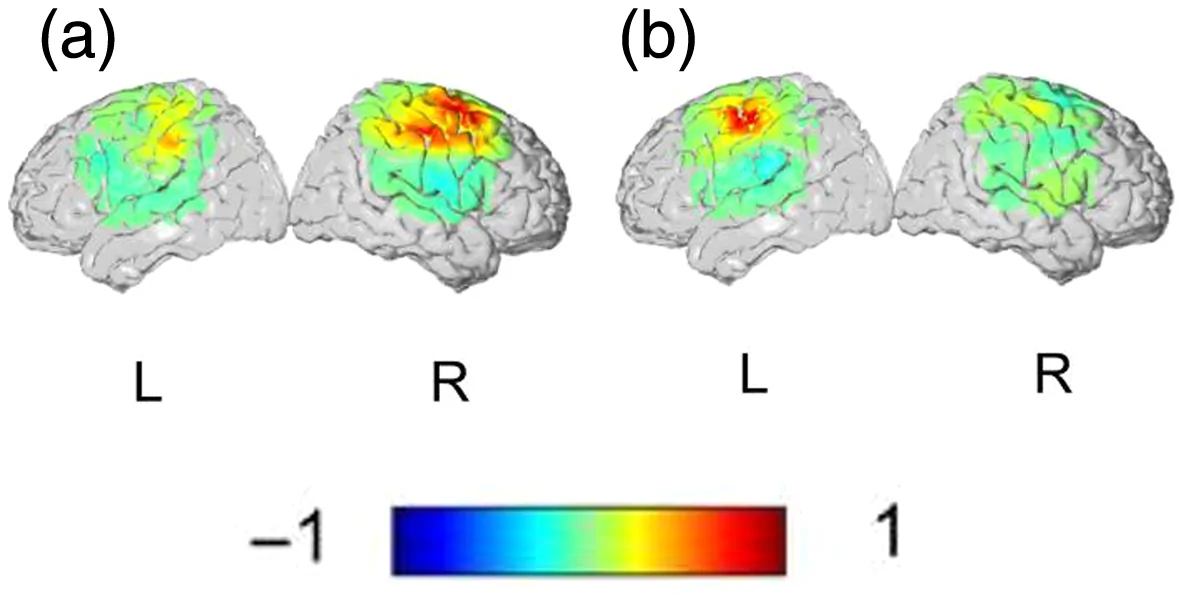
Validation of motor lateralization.^25^ The figure shows the group-averaged HbO during the ball-squeezing task. (a) Squeezing the left hand induces an increase in HbO in the right motor cortex. (b) Squeezing the right hand induces an increase in HbO in the left motor cortex. The color bar is normalized by the maximum HbO value. Red indicates HbO increase, blue indicates HbO decrease, and green indicates minimal HbO change.

### 5.4 Motion-Artifact Induction Task Validation

Figure 10 presents the PSC at 760 and 850 nm wavelengths and HbO/HbR concentration changes across the six motion types. Across all metrics, tilting up produced the highest and saying hello produced the lowest PSC and HbO/HbR concentration changes (Tables 2 and 3). The standard deviation of HbO and HbR concentration changes across the entire task is reported in Fig. S2 in the Supplementary Material.

**Fig. 10:**
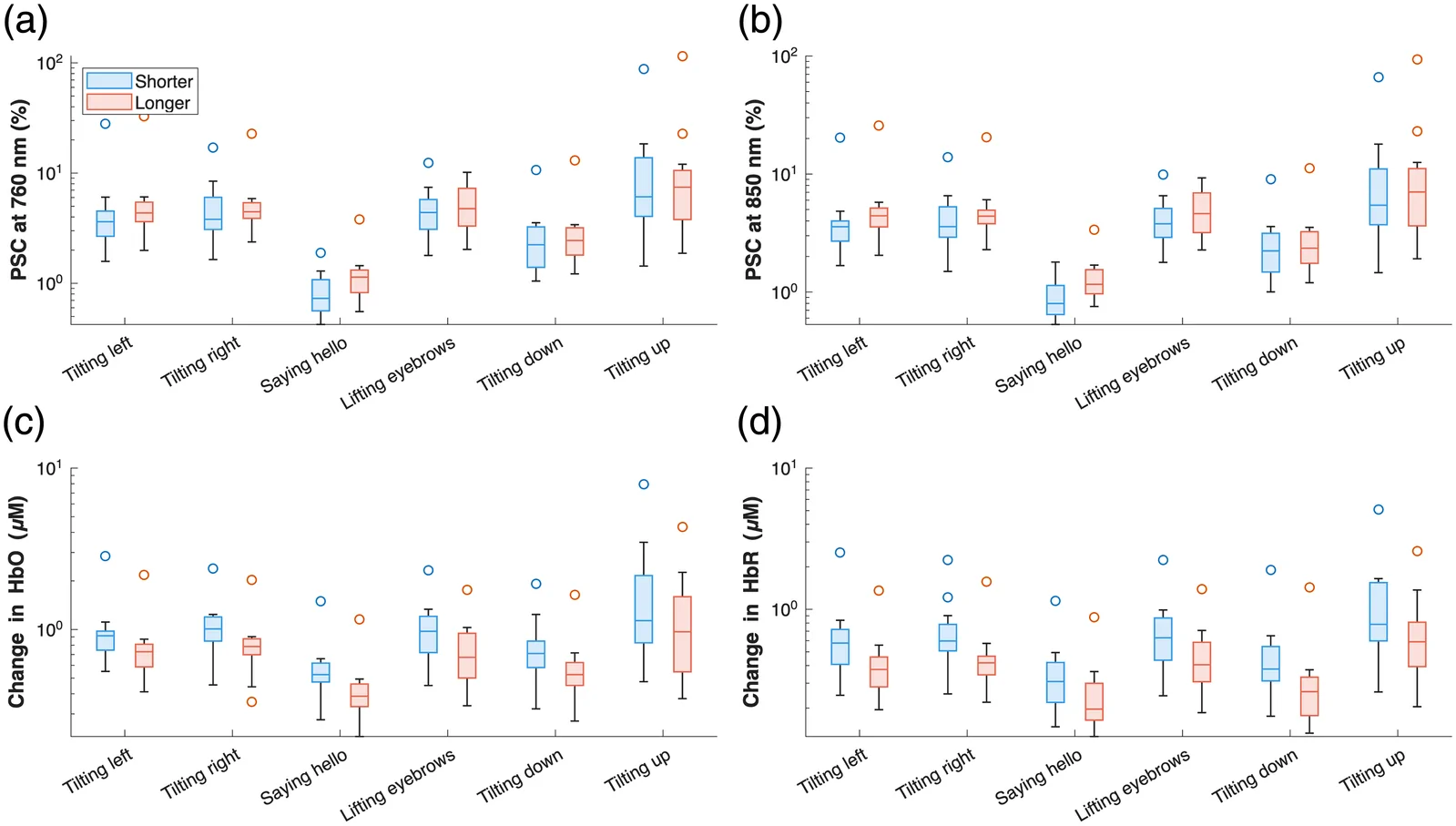
Percent signal change (PSC) and HbO/HbR changes across six motion types. (a) PSC at 760 nm and (b) PSC at 850 nm for shorter (blue) and longer (red) channels. (c) Change in HbO and (d) change in HbR for shorter (blue) and longer (red) channels. Boxplots are displayed with logarithmic y-axes. Central lines indicate median value, the upper and lower edges of the boxes represent the interquartile range (25th and 75th percentiles), the whiskers indicate the data range excluding outliers (defined as values extending beyond 1.5 × the interquartile range), and the outliers are marked by dots.

**Table 2:** Median percent signal change (PSC) (%) for shorter and longer channels at 760 and 850 nm wavelengths across six motion types.

	760 nm		850 nm	
	Shorter	Longer	Shorter	Longer
Tilting left	3.62	4.35	3.57	4.43
Tilting right	3.81	4.46	3.58	4.40
Saying hello	0.73	1.14	0.80	1.16
Lifting eyebrows	4.39	4.75	3.78	4.62
Tilting down	2.24	2.44	2.23	2.35
Tilting up	6.10	7.45	5.44	7.04

**Table 3:** Median values of changes in HbO and HbR (μM) for shorter and longer channels across six motion types.

	HbO		HbR	
	Shorter	Longer	Shorter	Longer
Tilting left	0.91	0.73	0.58	0.38
Tilting right	1.01	0.78	0.60	0.42
Saying hello	0.53	0.39	0.31	0.20
Lifting eyebrows	0.97	0.67	0.63	0.41
Tilting down	0.71	0.53	0.38	0.26
Tilting up	1.13	0.97	0.78	0.59

Figure 11 and Table 4 present the acceleration changes at Cz in X, Y, and Z axes across the six motion types. Head motion (tilting left, right, up, and down) produced larger acceleration changes than facial motions (saying hello, lifting eyebrows) in all three axes.

**Fig. 11:**
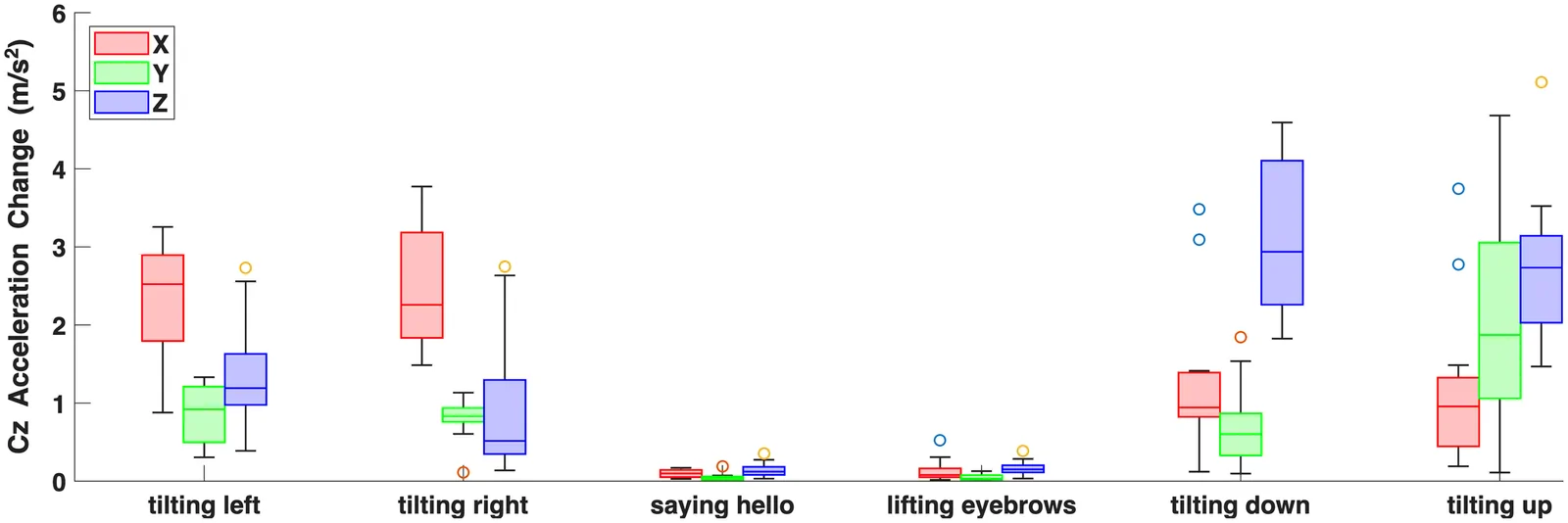
Acceleration changes ($m/s^{2}$) at Cz across six motion types. Acceleration changes are shown for X (red), Y (green), and Z (blue) axes. Central lines indicate median value, the upper and lower edges of the boxes represent the interquartile range (25th and 75th percentiles), the whiskers indicate the data range excluding outliers (defined as values extending beyond 1.5× the interquartile range), and the outliers are marked by dots.

**Table 4:** Median values of acceleration changes ($m/s^{2}$) at Cz for X, Y, Z axes across six motion types.

	X-axis	Y-axis	Z-axis
Tilting left	2.52	0.92	1.19
Tilting right	2.26	0.83	0.52
Saying hello	0.10	0.04	0.12
Lifting eyebrows	0.08	0.03	0.14
Tilting down	0.94	0.60	2.94
Tilting up	0.96	1.87	2.73

Finally, Spearman’s rank correlations between PSC and Cz accelerometer changes across both wavelengths and both channel distances were ρ=0.451 to 0.481 (Y axis), 0.313 to 0.354 (X axis), and 0.237 to 0.284 (Z axis) [Fig. 12].

**Fig. 12:**
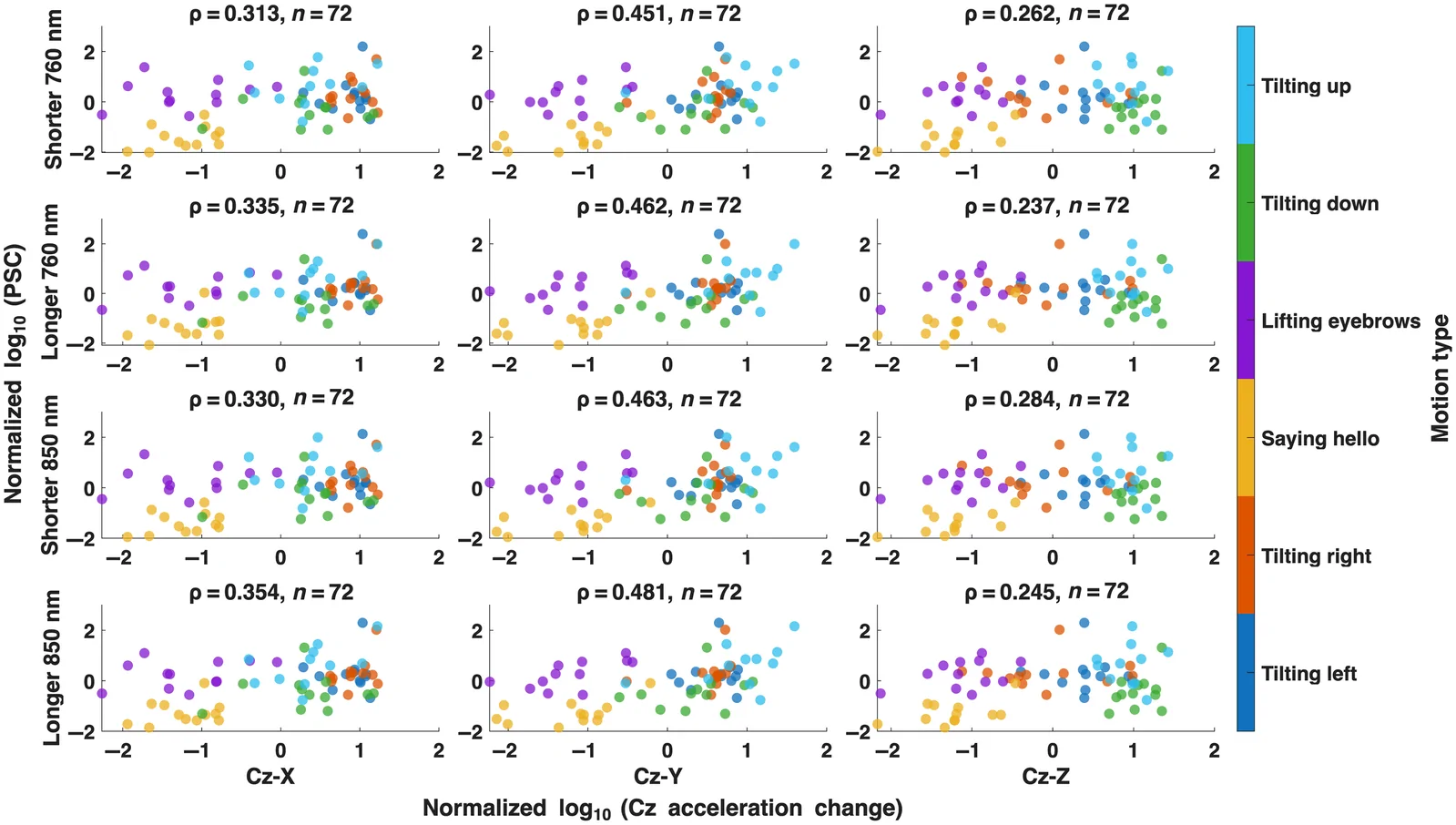
Spearman’s rank correlation coefficients between percent signal change (PSC) and Cz acceleration changes across six motion types. Each scatter plot displays normalized log-transformed PSC (Y-axis) against normalized log-transformed Cz acceleration change (X-axis). Spearman’s ρ is displayed above each panel. Rows represent channel distance and wavelength combinations. Columns represent the three acceleration axes. Each data point represents the mean value of one motion type from one participant (12 participants × 6 motion types, n=72). Colored dots indicate motion types.

## 6 Discussion and Conclusion

We introduced an open-access, multidistance fNIRS dataset collected from 12 healthy young adults during resting state, ball-squeezing, and motion-artifact induction tasks. Existing open-access motor task fNIRS datasets are primarily based on sparse fNIRS arrays. To our knowledge, no open-access multidistance fNIRS dataset was previously available for motor and motion-artifact induction tasks. We validated this dataset across signal noise level, functional integrity, and motion-artifact induction task characterization.

For signal noise level, the motion-artifact induction task produced significantly lower SNR and higher MAR than the other two tasks, indicating that the instructed movements induced motion artifacts. During resting state, shorter channels exhibited significantly higher SNR than longer channels. This difference is expected because increasing the SDS increases the probability of light absorption and decreases the light received by the detector, leading to SNR deterioration.^43^^,^^44^

For functional integrity, the ball-squeezing task elicited contralateral HbO increases in the motor cortex. No significant differences in SNR or MAR were found between the resting state and ball-squeezing tasks, indicating comparable signal quality across the two conditions.

For the motion-artifact induction task characterization, tilting up produced the largest PSC and HbO/HbR changes while saying hello produced the smallest. Accelerometer data demonstrated that head-tilting motions produced larger acceleration changes than facial motions. Correlations between PSC and accelerometer changes were inconsistent across all 12 combinations (2 wavelengths × 2 channel distances × 3 acceleration axes), suggesting that the relationship between head kinematics and optical signal changes varies by acceleration axis.

Several limitations should be considered. First, the sample included only 12 healthy young adults, predominantly White (10 of 12), which limits demographic variability, generalizability, and the robustness of benchmarking based on this dataset. Second, the fixed task order may have introduced confounds from fatigue or optode displacement over time. However, the c.v. values remained stable across the three ball-squeezing runs (Fig. S1 in the Supplementary Material), suggesting that signal quality did not change across runs. Third, external behavioral force was not measured in the ball-squeezing task, so motor output cannot be quantified or correlated with hemodynamic responses. Fourth, the probe covered only bilateral motor cortex, which limits the applicability of this dataset to other brain regions. Fifth, no individual structural MRI was acquired. Image reconstruction may involve localization error from inter-subject anatomical variability. Despite this error, atlas-guided DOT can achieve reasonable anatomical localization without subject-specific MRI.^45^ Sixth, the functional validation reproduced results (Fig. 9) from our previous study^25^ rather than performing an independent reconstruction.

Despite these limitations, this dataset could support several uses. The multidistance fNIRS data with overlapping measurements can be used to test image reconstruction algorithms, such as regularization. Supplementary Material S1 provides the preprocessing, sensitivity matrix, and reconstruction pipeline for reproduction. The shorter (19 mm) channels include superficial and cortical sensitivity. Using them as regressors could remove systemic physiological noise and part of the cortical response from longer channels,^29^ enabling evaluation of this trade-off in short-separation regression methods. The noise-level validation across three tasks provides reference values for signal-quality assessment of this dataset. The ball-squeezing task, with defined event timing and expected contralateral activation across three runs, can serve as a benchmark for GLM-based analysis pipelines. The ball-squeezing data can also be used for brain–computer interface classification. The fNIRS and accelerometer data from the motion-artifact induction task provide benchmarking for motion-correction algorithms.

## Supplementary Material

10.1117/1.NPh.13.S3.S32606.s01

## Data Availability

The data presented in this article are publicly available in OpenNeuro at https://doi.org/10.18112/openneuro.ds007420.v1.0.0. The custom MATLAB scripts developed for technical validation and figure generation are available at https://github.com/Shibo3697/Validation_SNR_MotionArtifact.git.
