# Supplementary material for: Open-access multidistance fNIRS dataset generated during ball-squeezing and motion-artifact induction tasks
Source: Neurophotonics. 2026 Oct 22;13(Suppl 3):S32606. doi: 10.1117/1.NPh.13.S3.S32606 (PMC13696112; doi:10.1117/1.NPh.13.S3.S32606)
Supplement: Supplementary file 1 [file NPh_013_S32606_SD001.pdf]

Supplementary information

Image Reconstruction Processing Pipeline

The functional validation in Section 4.3 followed the preprocessing and image reconstruction method described in paper "Short-separation regression incorporated diffuse optical tomography image reconstruction modeling for high-density functional near-infrared spectroscopy". This supplement summarizes the processing steps for reproduction.

Preprocessing

We first pruned noisy channels with signal-to-noise ratio below 5 using the hmrR_PruneChannels function in Homer3 and then converted light intensity to changes in optical density. Motion artifacts were corrected using the SplineSG method (hmrR_MotionCorrectSplineSG in Homer3, interpolation parameter = 0.99).

Sensitivity Matrix

We computed the sensitivity matrix for two wavelengths (760 nm and 850 nm) using Monte Carlo simulations (MCXLAB toolbox, 10^7 photons per optode). The simulations used the Colin27 head model segmented into five tissue types: scalp, skull, CSF, gray matter, and white matter using SPM software. The absorption coefficients μ_a and reduced scattering coefficients μ'_s are listed in Table S1.

Table S1. Absorption coefficients μ_a and reduced scattering coefficients μ'_s for different tissue types.

**Tissue**	**μ_a (mm^{-1}) 760 nm**	**μ_a (mm^{-1}) 850 nm**	**μ'_s (mm^{-1}) 760 nm**	**μ'_s (mm^{-1}) 850 nm**
Scalp	0.0177	0.0190	0.73	0.64
Skull	0.0125	0.0139	0.93	0.84
CSF	0.0021	0.0040	0.30	0.30

Gray matter	0.0195	0.0192	1.18	0.67
White matter	0.0195	0.0208	1.18	1.01

Image Reconstruction

We applied the SS-DOT model, which simultaneously performs short-separation regression and diffuse optical tomography image reconstruction. The model was solved using Tikhonov regularization. Reconstructed images were generated at the time-to-peak of the hemodynamic response. Group-averaged HbO images were computed across all 12 subjects. The color bar was normalized by the maximum HbO value (Fig. 9).

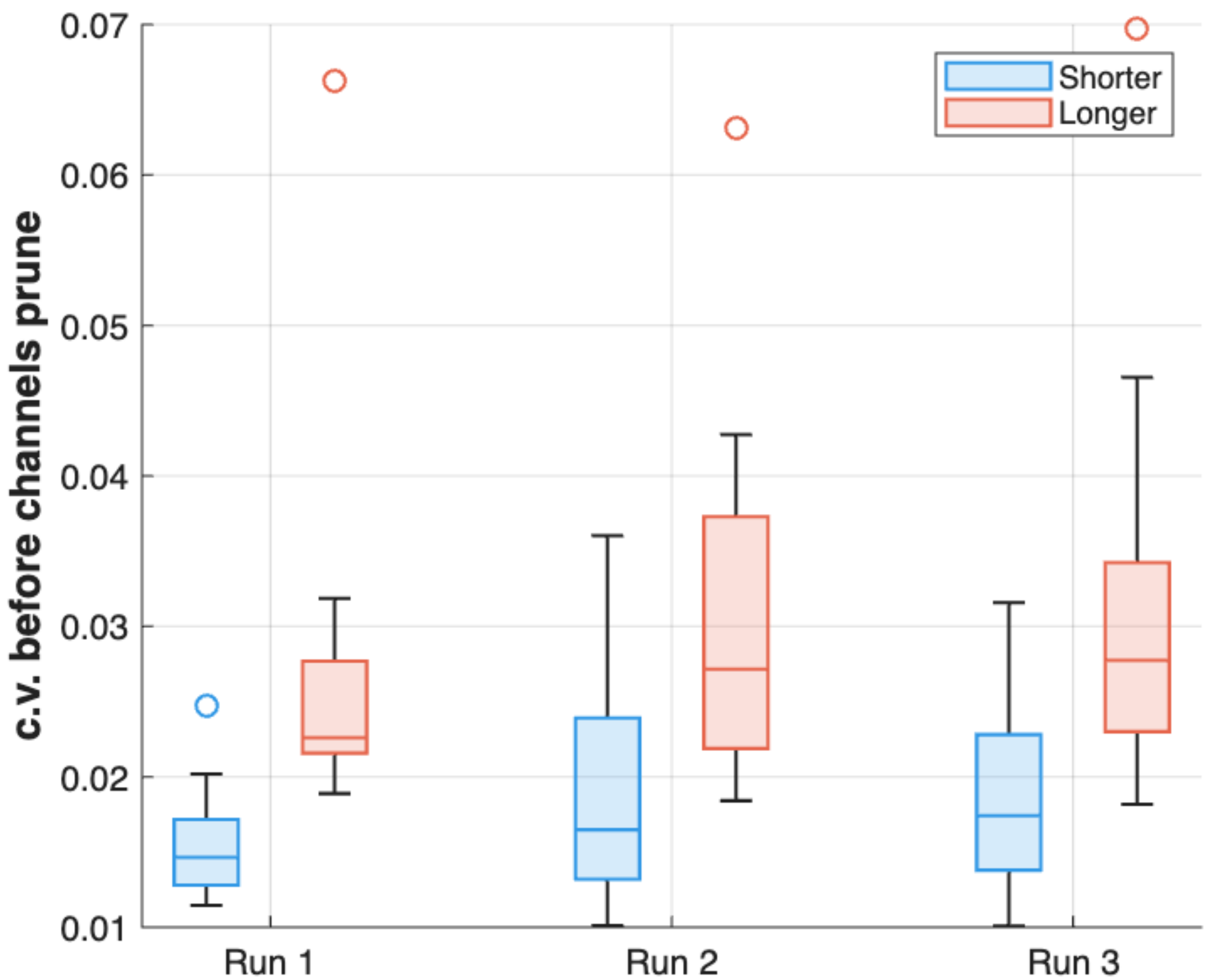

Figure S1. Coefficient of variation (c.v.) before channel pruning across three runs of the ball-squeezing task for shorter (blue) and longer (red) channels.

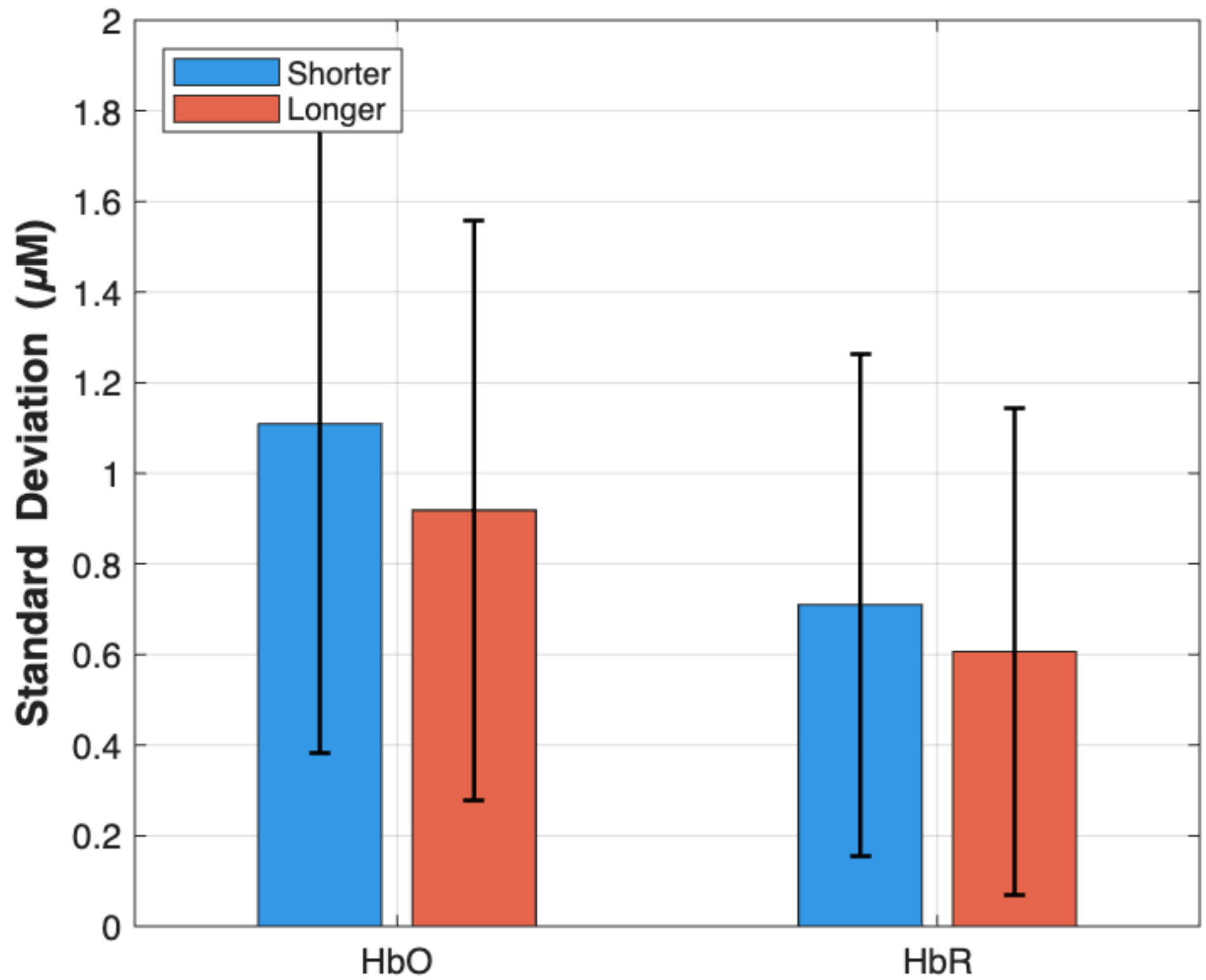

Figure S2. Standard deviation of the changes in HbO and HbR during the entire motion-artifact induction task.

The scalp coupling index (SCI) was computed using the QT-NIRS toolbox. SCI measures the cross-correlation between the two-wavelength signals within the cardiac frequency band, where higher values indicate better optode-scalp coupling. The peak spectral power (PSP) measures the relative power of the cardiac frequency peak in the signal's power spectrum. A channel was classified as high quality if SCI ≥ 0.8 and PSP ≥ 0.1 for at least 90% of the recording time.

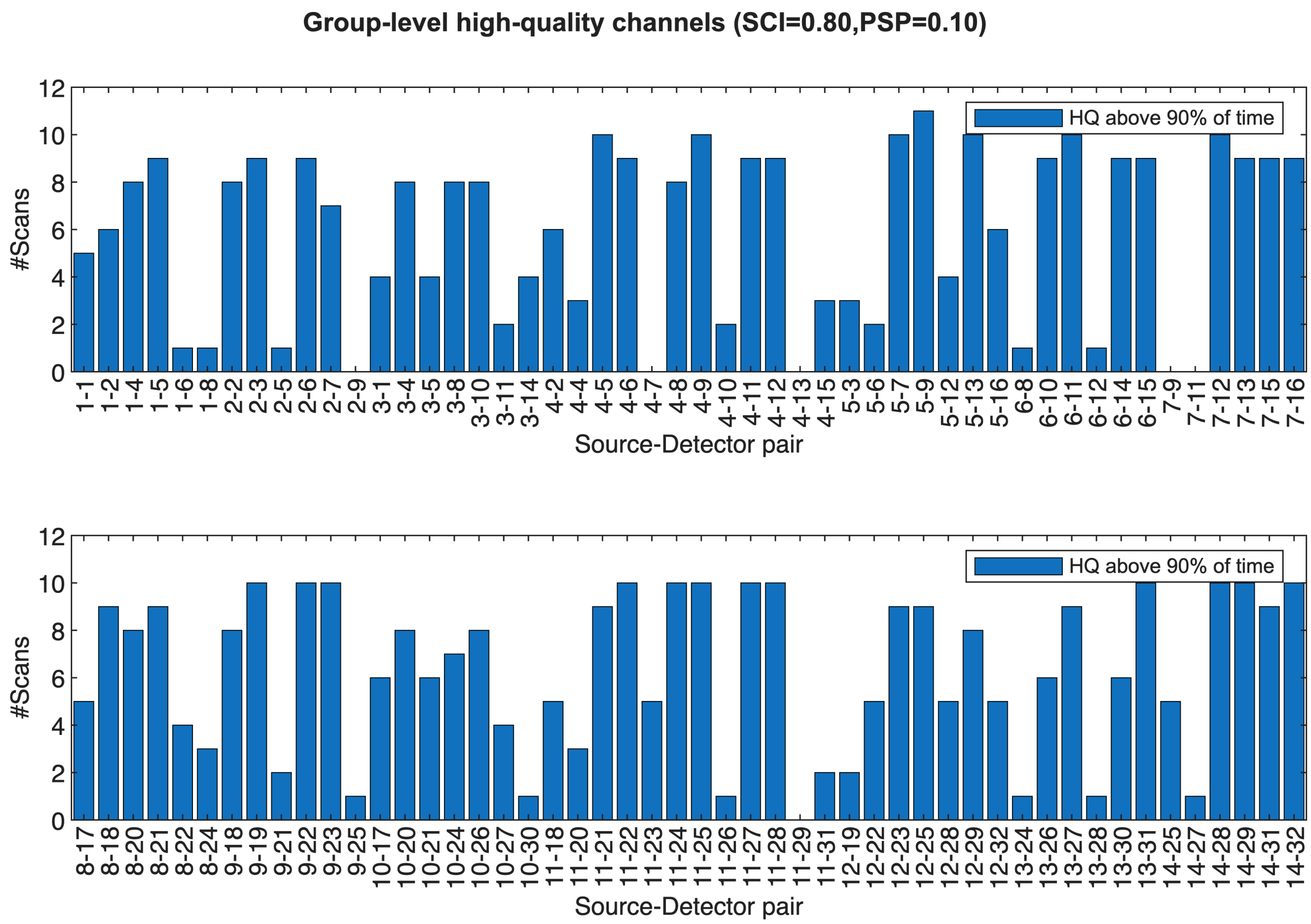

Figure S3. SCI high-quality channels for resting state.

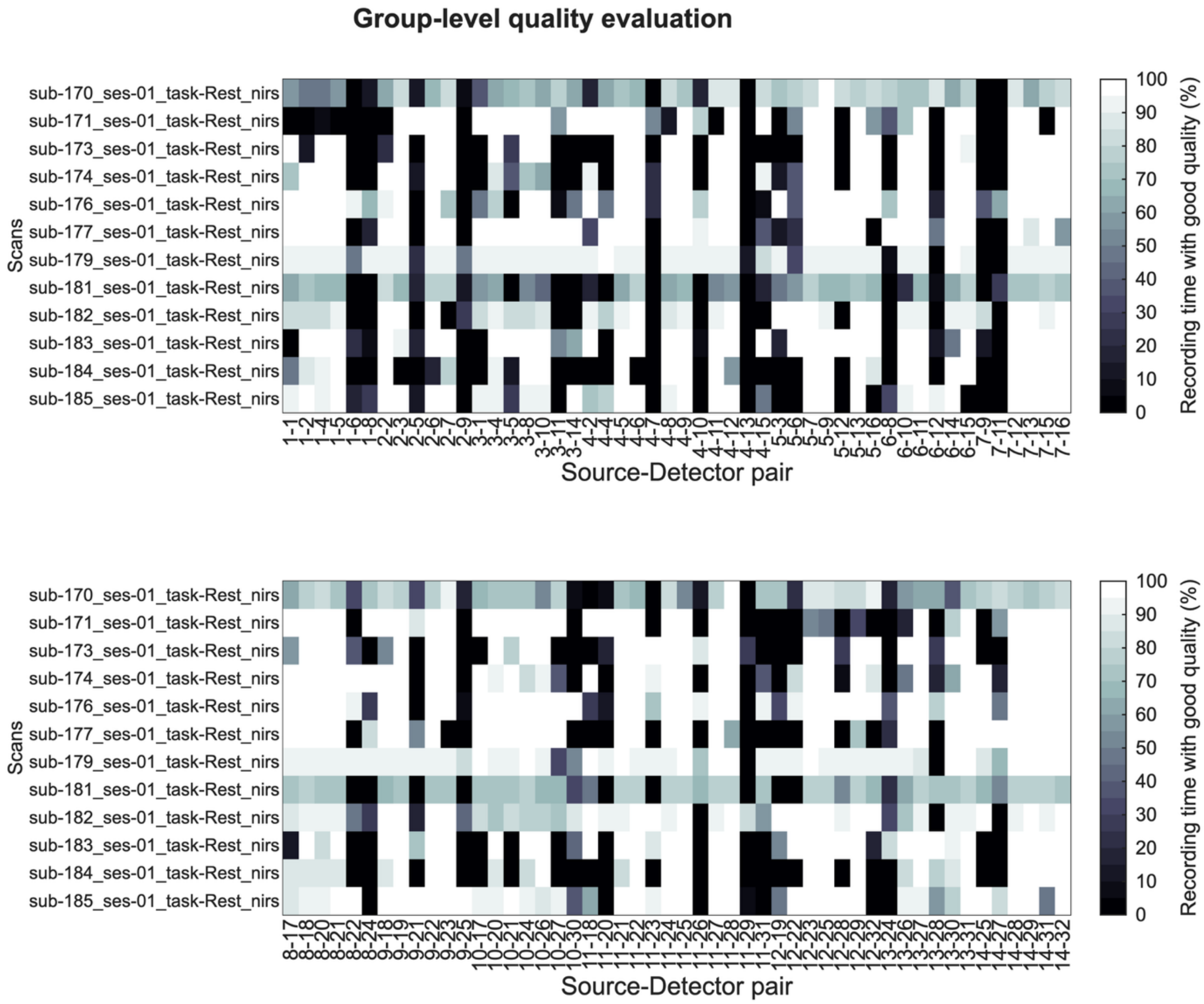

Figure S4. SCI quality evaluation for resting state.

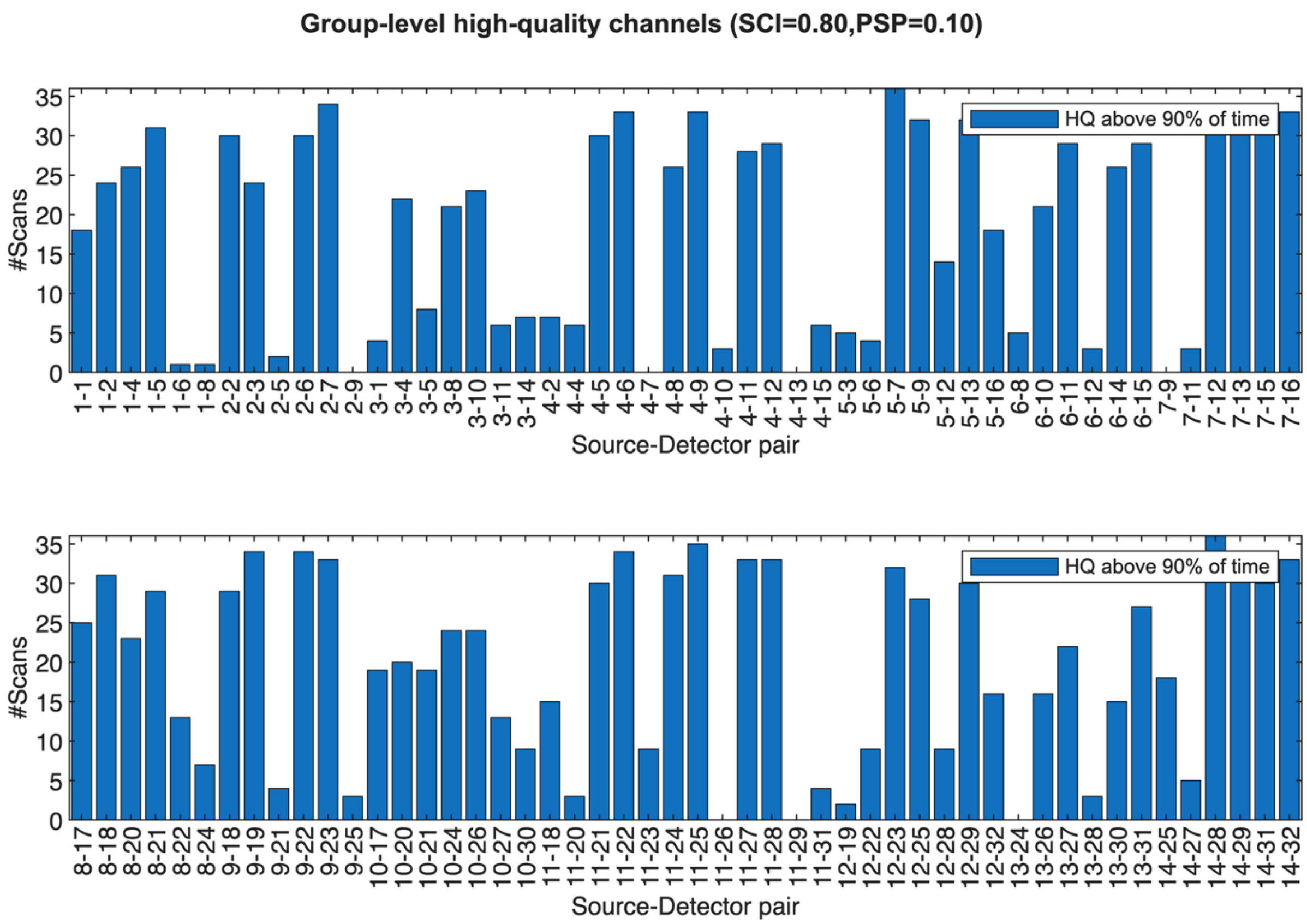

Figure S5. SCI high-quality channels for ball-squeezing.

Group-level quality evaluation

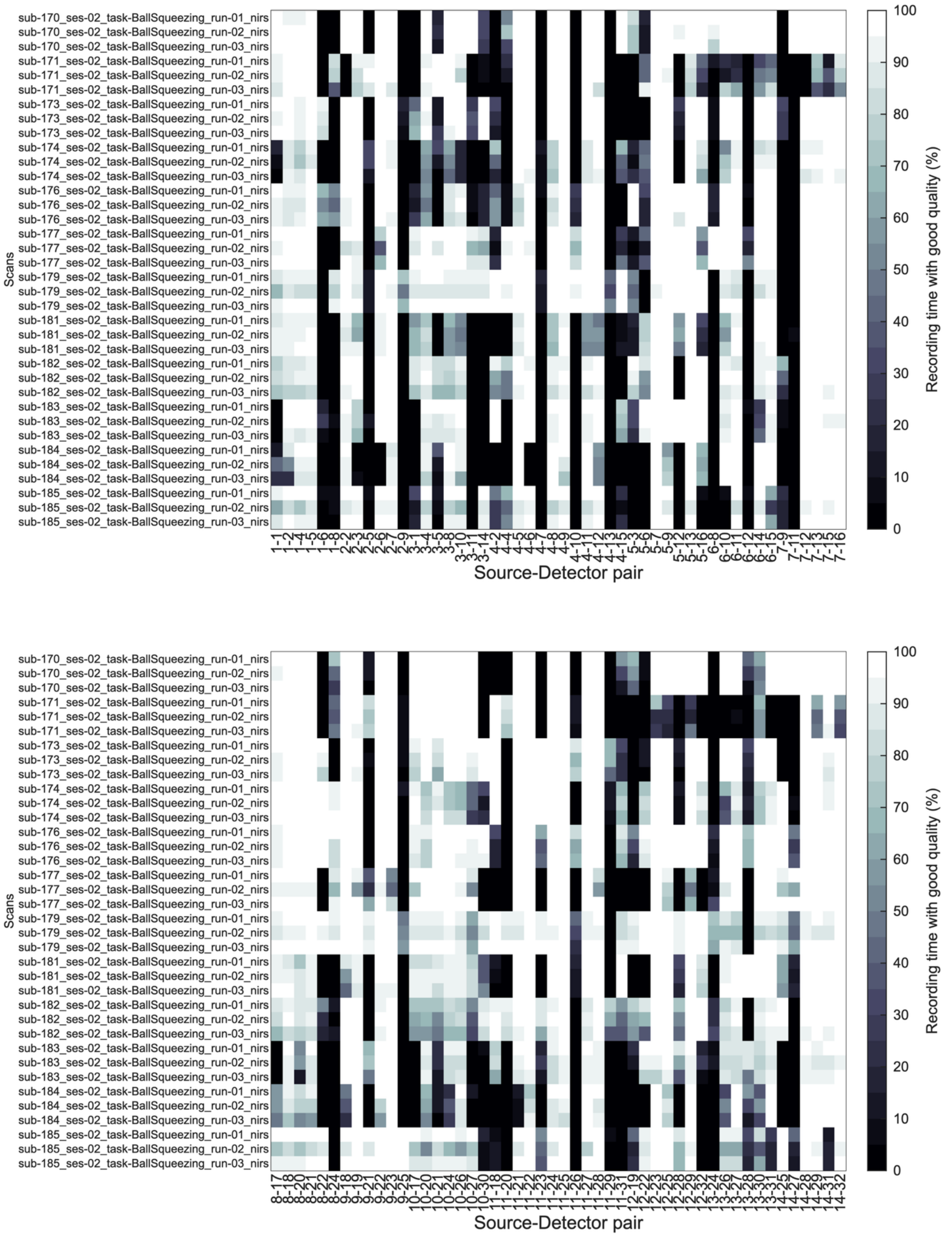

Figure S6. SCI quality evaluation for ball-squeezing.

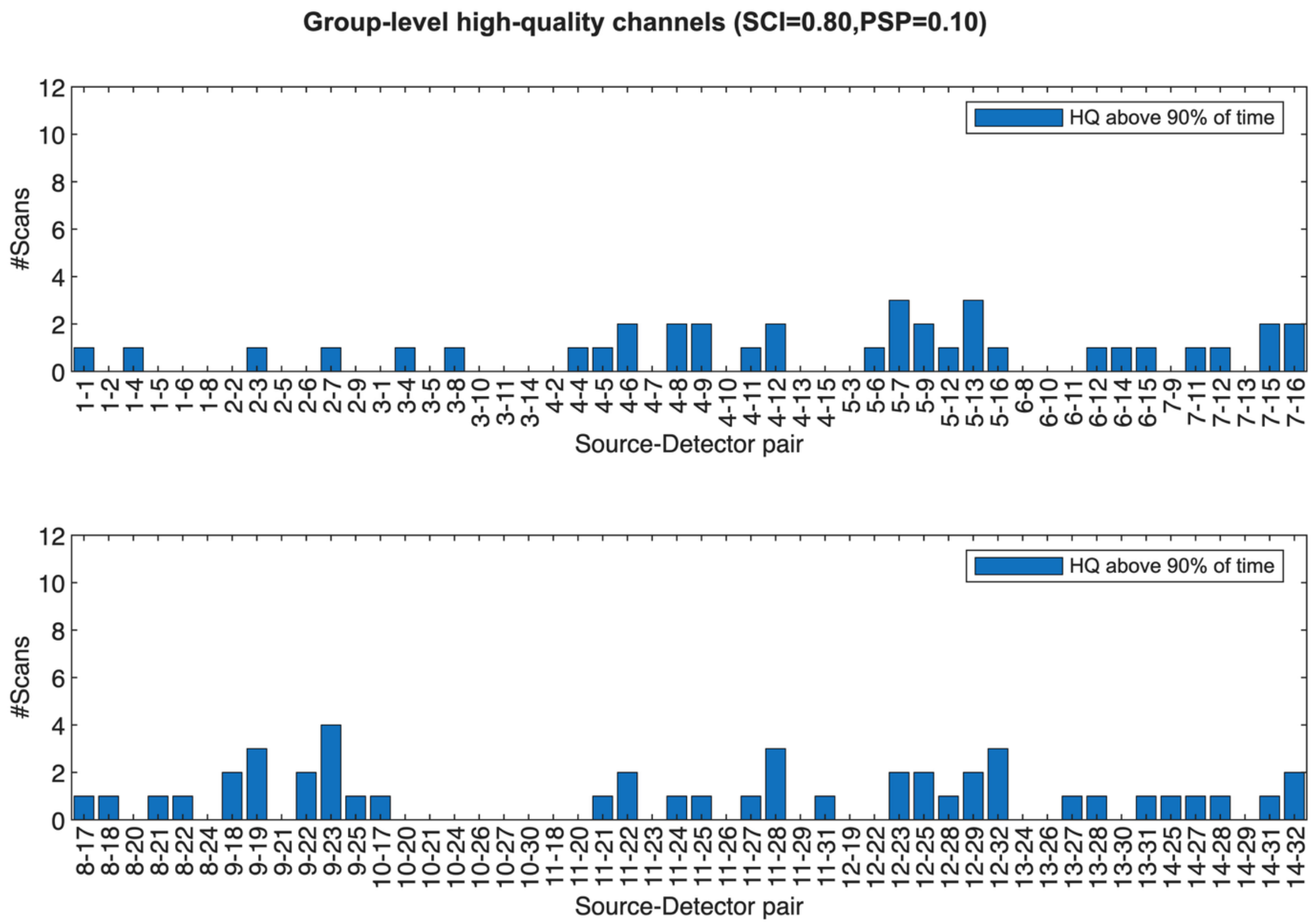

Figure S7. SCI high-quality channels for motion-artifact induction.

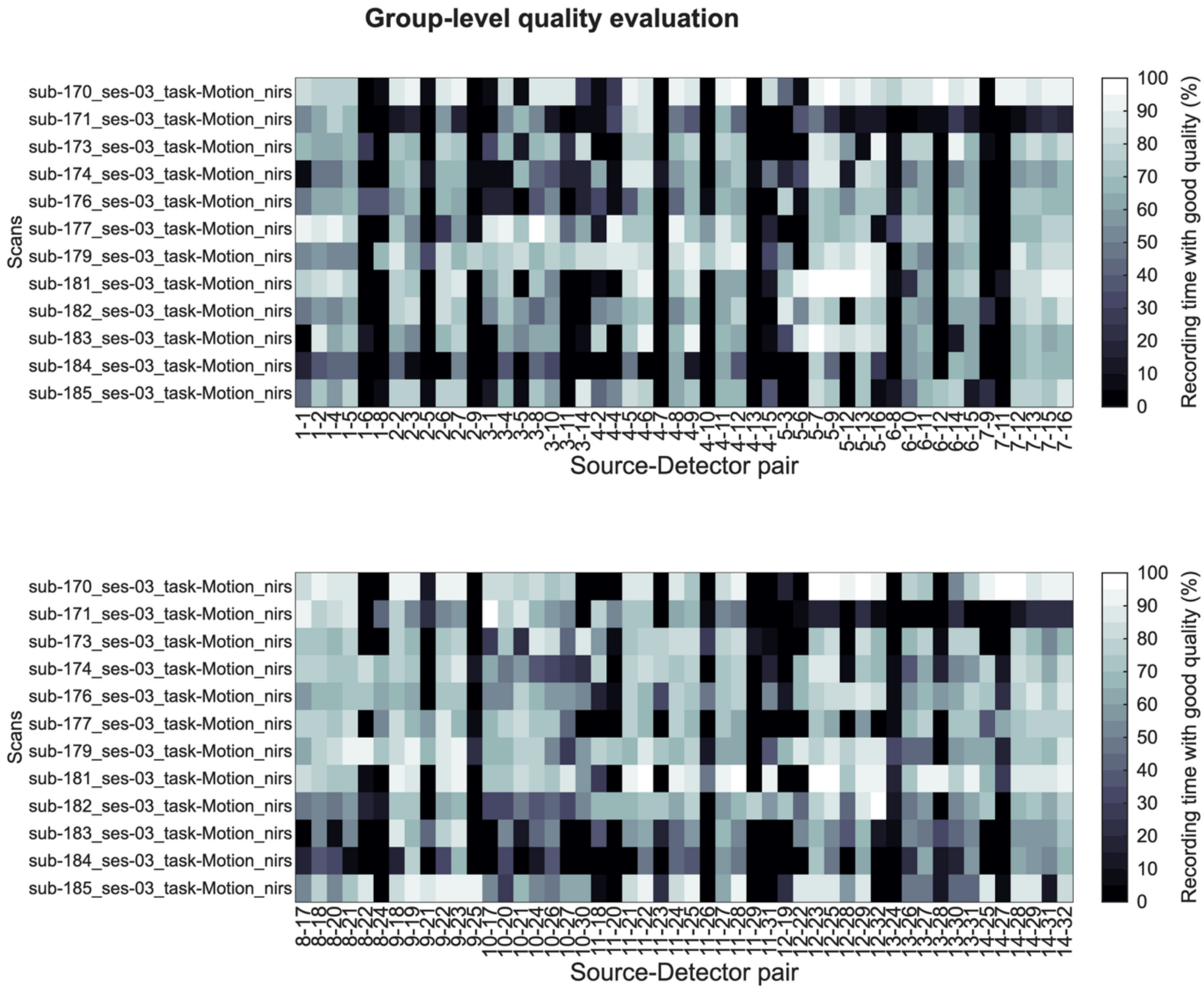

Figure S8. SCI quality evaluation for motion-artifact induction.

BIDS Validation

To confirm BIDS compliance, the dataset was validated using the BIDS Validator (v3.0.0-alpha.3). The validator returned no errors across all 459 files. Warnings are listed in Table S2 and do not affect dataset compliance.

Table S2. BIDS Validator warnings.

**Warning**
UNKNOWN_BIDS_VERSION
README_FILE_MISSING
JSON_KEY_RECOMMENDED (HEDVersion)
JSON_KEY_RECOMMENDED (GeneratedBy)

JSON_KEY_RECOMMENDED (SourceDatasets)
JSON_KEY_RECOMMENDED (NIRSCoordinateProcessingDescription)
JSON_KEY_RECOMMENDED (FiducialsCoordinates)
JSON_KEY_RECOMMENDED (FiducialsCoordinateUnits)
JSON_KEY_RECOMMENDED (FiducialsCoordinateSystem)
JSON_KEY_RECOMMENDED (AnatomicalLandmarkCoordinateSystem)
JSON_KEY_RECOMMENDED (AnatomicalLandmarkCoordinateUnits)
JSON_KEY_RECOMMENDED (AnatomicalLandmarkCoordinateSystemDescription)
JSON_KEY_RECOMMENDED (FiducialsCoordinateSystemDescription)
TSV_COLUMN_TYPE_REDEFINED (x)
TSV_COLUMN_TYPE_REDEFINED (y)
TSV_COLUMN_TYPE_REDEFINED (z)
TSV_COLUMN_TYPE_REDEFINED (onset)
TSV_COLUMN_TYPE_REDEFINED (duration)
EVENT_ONSET_ORDER
SIDECAR_KEY_RECOMMENDED (StimulusPresentation)
SIDECAR_KEY_RECOMMENDED (Manufacturer)
SIDECAR_KEY_RECOMMENDED (ManufacturersModelName)
SIDECAR_KEY_RECOMMENDED (SoftwareVersions)
SIDECAR_KEY_RECOMMENDED (DeviceSerialNumber)
SIDECAR_KEY_RECOMMENDED (RecordingDuration)
SIDECAR_KEY_RECOMMENDED (HeadCircumference)
SIDECAR_KEY_RECOMMENDED (HardwareFilters)
SIDECAR_KEY_RECOMMENDED (SubjectArtefactDescription)
SIDECAR_KEY_RECOMMENDED (TaskDescription)
SIDECAR_KEY_RECOMMENDED (Instructions)
SIDECAR_KEY_RECOMMENDED (CogAtlasID)
SIDECAR_KEY_RECOMMENDED (CogPOID)
SIDECAR_KEY_RECOMMENDED (InstitutionName)
SIDECAR_KEY_RECOMMENDED (InstitutionAddress)
SIDECAR_KEY_RECOMMENDED (InstitutionalDepartmentName)
SIDECAR_KEY_RECOMMENDED (CapManufacturer)
SIDECAR_KEY_RECOMMENDED (CapManufacturersModelName)
SIDECAR_KEY_RECOMMENDED (SourceType)
SIDECAR_KEY_RECOMMENDED (DetectorType)
SIDECAR_KEY_RECOMMENDED (ShortChannelCount)
SIDECAR_KEY_RECOMMENDED (NIRSPlacementScheme)
